# Supplementary figures and images for: Light intensity and opsin sensitivity shape the morphology of cone photoreceptor outer segments
Source: PLoS Biol. 2026 Feb 18;24(2):e3003654. doi: 10.1371/journal.pbio.3003654 (PMC12915902; doi:10.1371/journal.pbio.3003654)

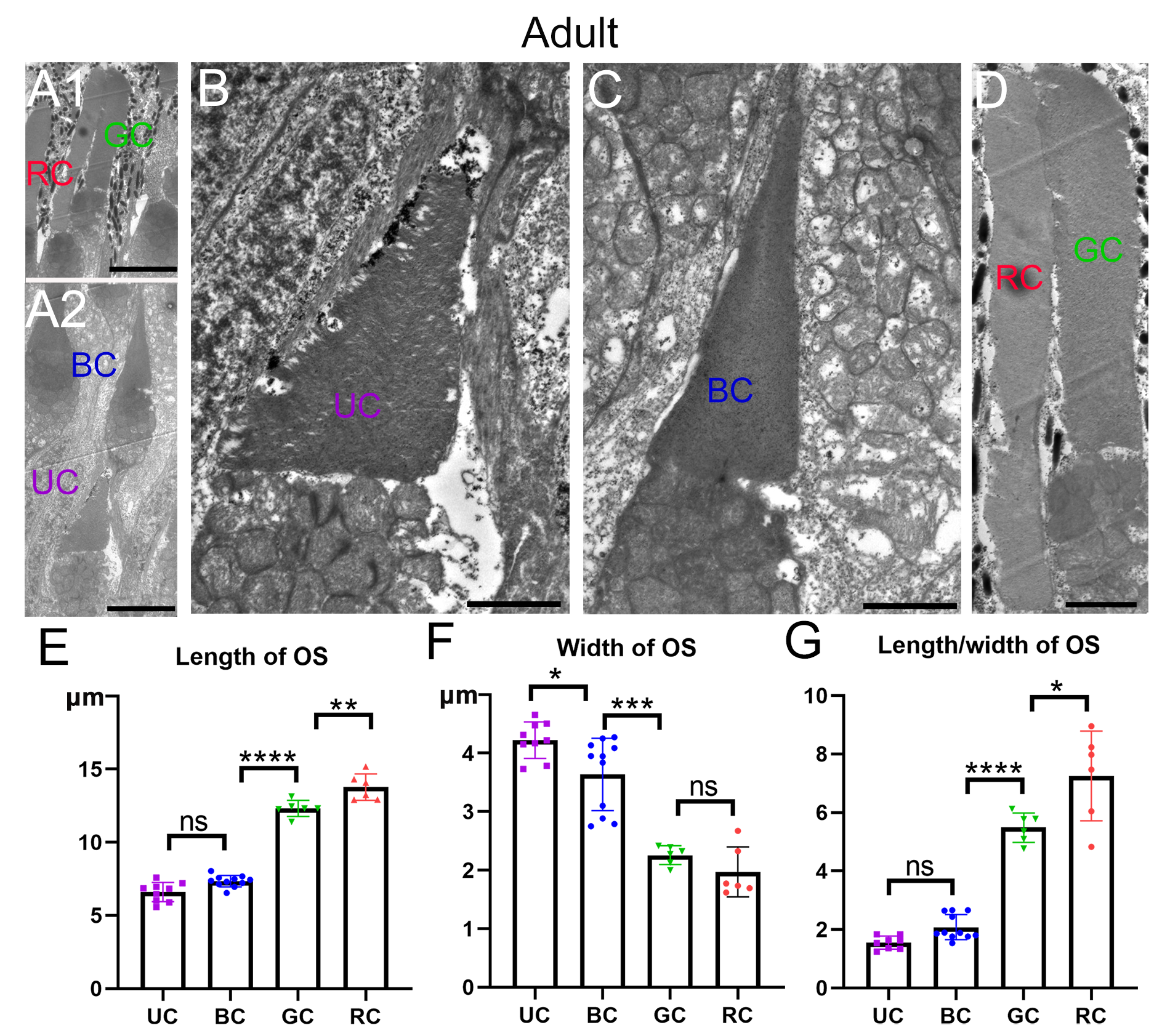

Supplement: S1 Fig — (A1, A2) Transmission electron micrograph (TEM) showing the distribution of cone OS in the adult zebrafish retina. (B–D) Higher magnification TEM images illustrating the ultrastructure of OS in UV cones (B), blue cones (C), and double cones (D). (E–G) Quantitative analyses of OS morphology. Abbreviations: RC, red cone; GC, green cone; BC, blue cone; UC, UV cone. Scale bars: 5 μm in (A); 2 μm in (B–D). Data information: In (E–G), each dot represents one photoreceptor OS. Sample sizes per group are as follows: OS n(UC) = 9, n(BC) = 12, n(GC) = 6, n(RC) = 6. Data were derived from N = 3 zebrafish per group. Statistical significance was determined by one-way ANOVA with Bonferroni’s post hoc test. p < 0.05; ** p < 0.01; **** p < 0.0001; ns, not significant. The data underlying this Figure can be found in S1 Data. (TIF) [file pbio.3003654.s001.tif]

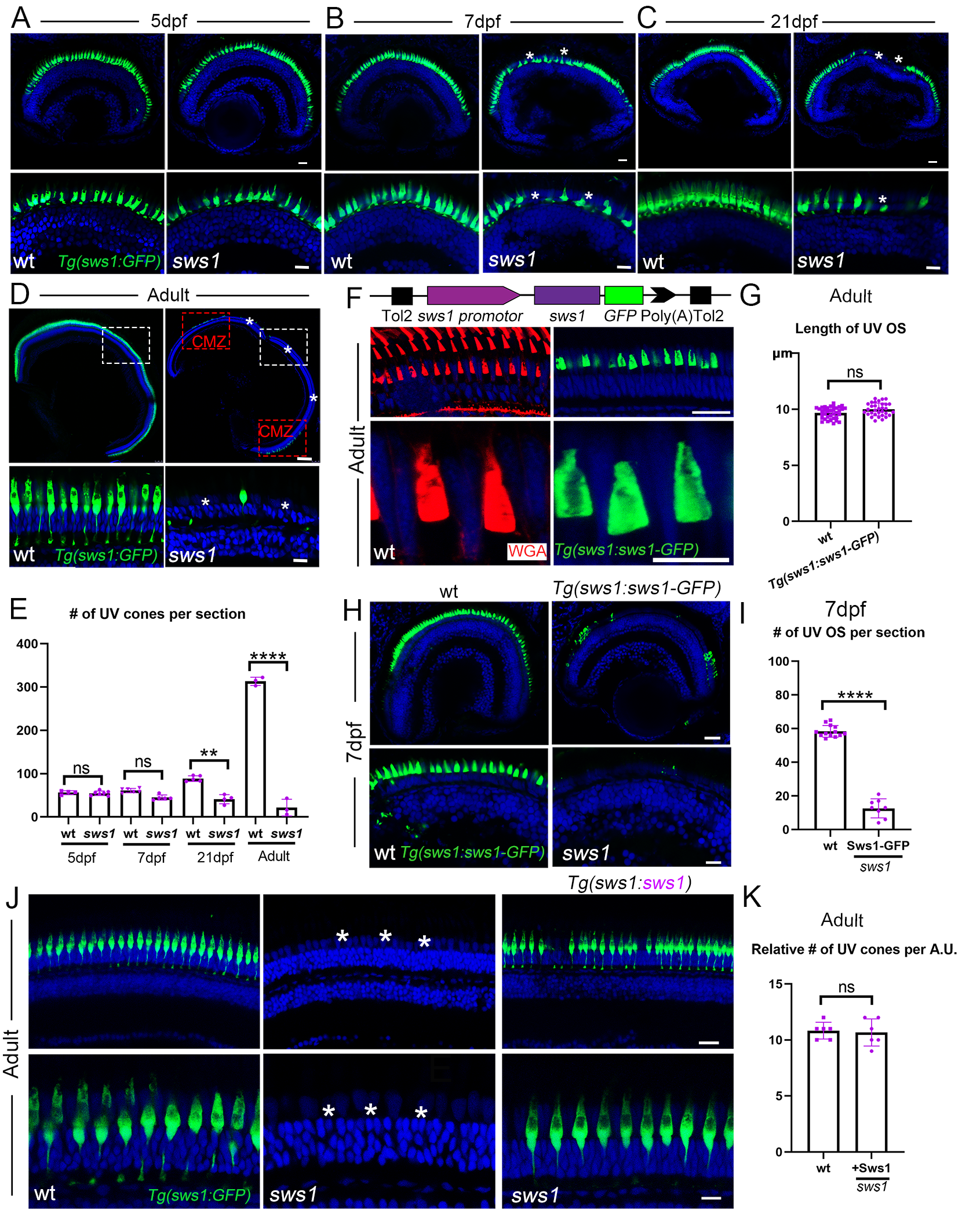

Supplement: S2 Fig — (A–D) Confocal images showing the morphology of UV cones in wild-type and sws1 mutant retinas at different developmental stages, as indicated. Enlarged views are shown at the bottom. UV cone cell bodies are labeled with Tg(sws1:GFP) (green). White asterisks indicate regions lacking UV cone cell bodies. In (D), the red rectangle outlines the remaining UV cones in the ciliary marginal zone (CMZ), and the white rectangle highlights a region shown in the enlarged view below. Adult = 4 months. (E) Quantitative analysis of UV cone cell numbers across different developmental stages. (F) Confocal images showing the morphology of UV cones in wild-type (labeled with WGA, red) and Tg(sws1:sws1-GFP) (green). (G) Quantification of UV cone OS length in adult zebrafish. (H) Representative confocal images showing the morphology of UV cones following exogenous expression of Sws1-GFP in the sws1 mutant background. (I) Quantitative analysis of the number of UV cone OS. (J) Confocal micrographs showing that expression of zebrafish Sws1 fully rescues UV cones in sws1 mutants. (K) Quantitative analysis of the number of UV cones in adult zebrafish. DAPI (blue) marks cell nuclei. Scale bars: 25 μm (low-magnification images); 10 μm (high-magnification images). Data information: In panels (E, I, K), each dot represents the number of UV cones from the section of a larvae or adult fish. We only collect one data for each sample. Sample sizes per group (E) are as follows: N(wt 5 dpf) = 4, N(sws1 5 dpf) = 7, N(wt 7 dpf) = 6, N(sws1 7 dpf) = 5, N(wt 21 dpf) = 5, N(sws1 21 dpf) = 4, N(wt 4 month) = 3, N(sws1 4 month) = 3. Statistical significance was determined by Kruskal–Wallis with Dunn’s post hoc test. In panel (I) Sample sizes per group are as follows: N(wt 7 dpf) = 17, N(Sws1-GFP 7 dpf) = 8. Statistical significance was determined by the Mann–Whitney test. In panel (K), sample sizes per group are as follows: N(wt) = 6, N(+Sws1) = 6. Statistical significance was determined by the Mann–Whitney t [file pbio.3003654.s002.tif]

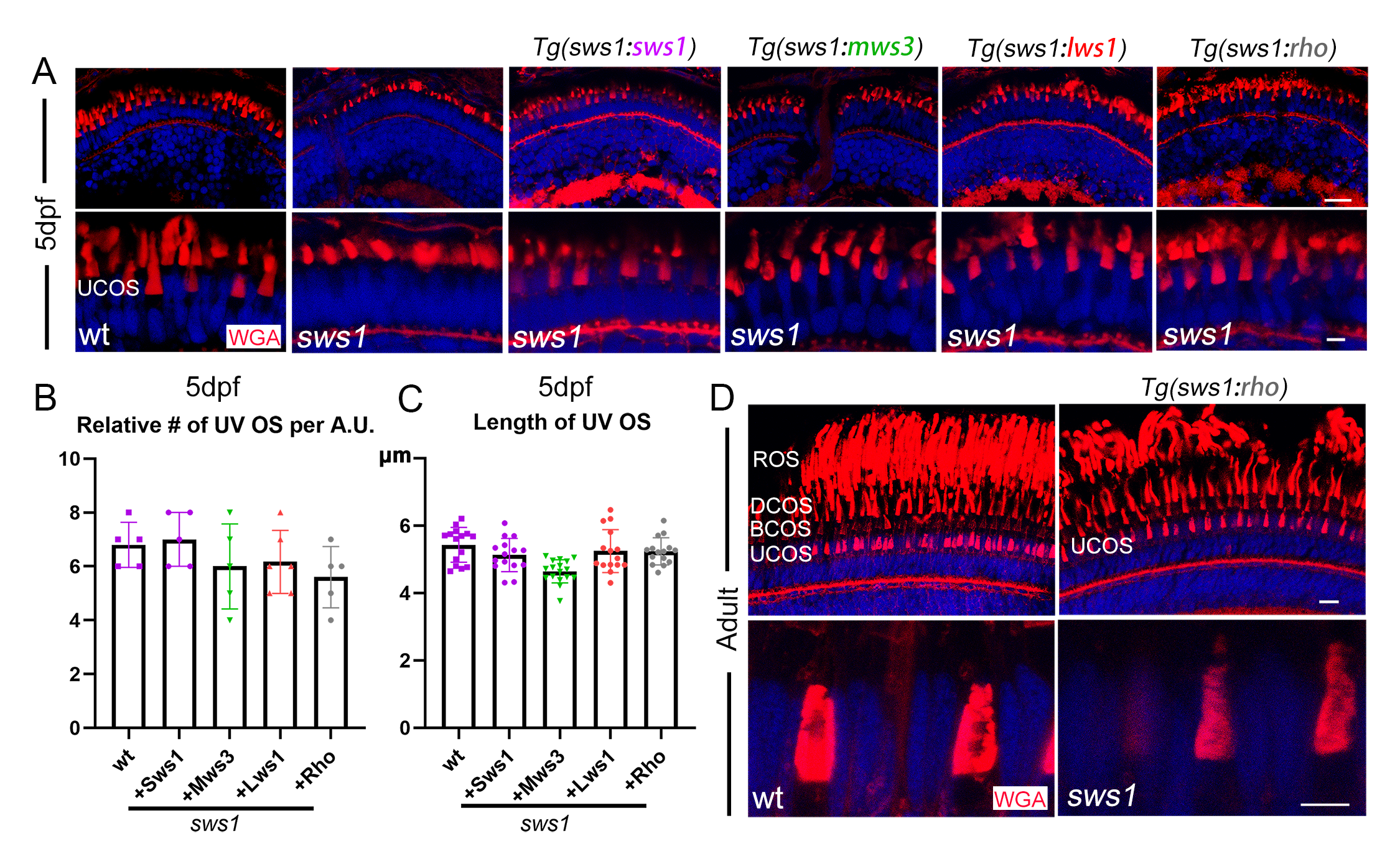

Supplement: S3 Fig — (A) Confocal images illustrating the morphology of UV cone OS in 5 dpf zebrafish with different transgenic backgrounds, as indicated. (B, C) Quantitative analysis of the number and length of UV cone OS in 5 dpf zebrafish larvae. (D) Confocal images showing the morphology of UV cone OS in adult zebrafish following ectopic expression of rhodopsin. The OS were labeled with WGA. DAPI (blue) marks cell nuclei. Scale bars: 15 μm (low-magnification) and 5 μm (high-magnification) in (A) and (D). Data information: In panel (B), each dot represents the relative number of UV cone OS from the section of a larvae. We only collect one data for each sample. Relative # of UV cone OS per A.U (arbitrary units) is calculated by the number of UV cone OS per arbituary length of confocal images (38.75 µm). Sample sizes per group are as follows: N(wt) = 5, N(+Sws1) = 5, N(+Mws3) = 5, N(+Lws1) = 6, N(+Rho) = 5. In panel (C), each dot represents one photoreceptor OS. Sample sizes per group are as follows: OS n(wt) = 15, n(+Sws1) = 15, n(+Mws3) = 17, n(+Lws1) = 15, n(+Rho) = 15. Data were derived from N = 3–6 independent biological replicates per group. The data underlying this Figure can be found in S1 Data. (TIF) [file pbio.3003654.s003.tif]

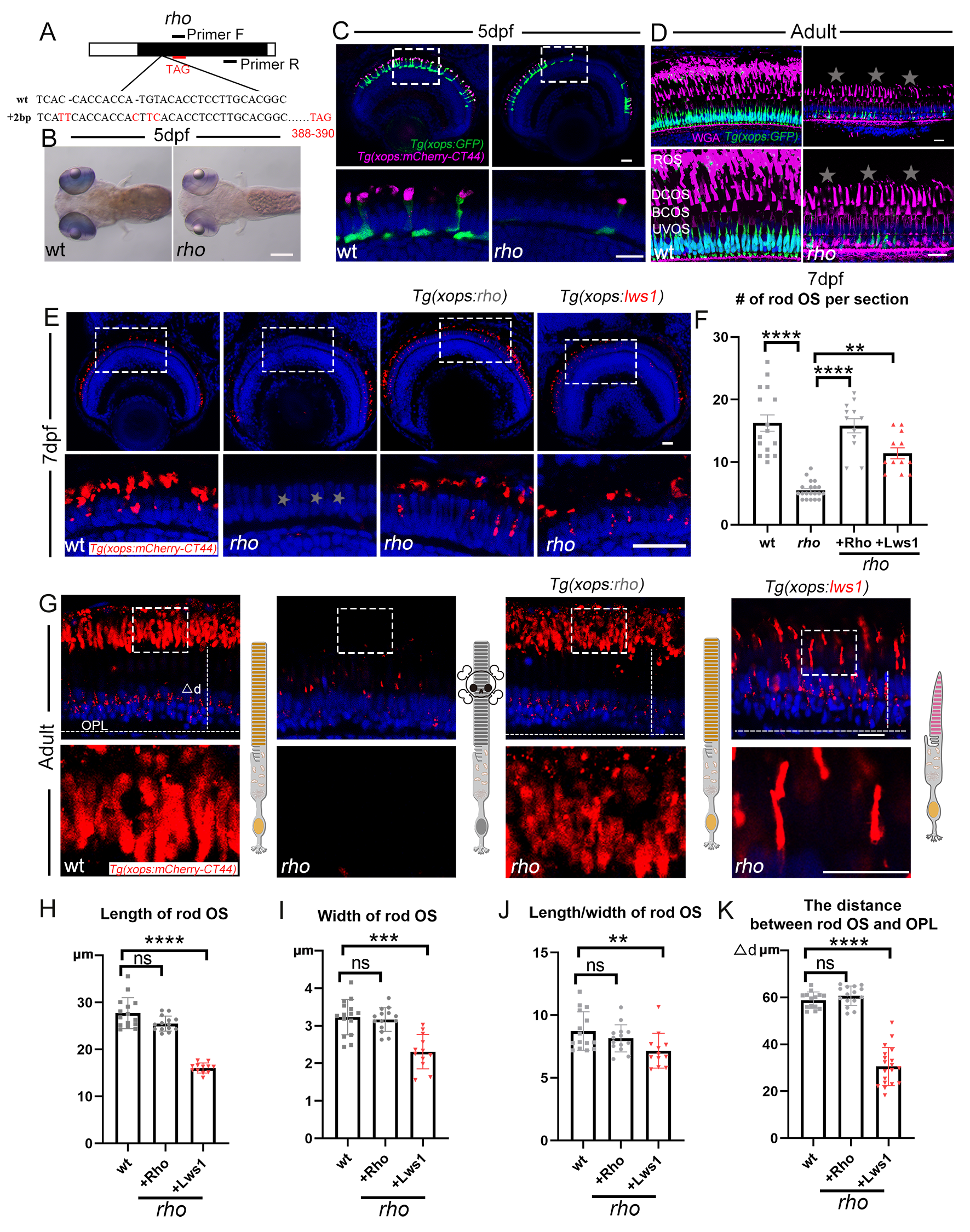

Supplement: S4 Fig — (A) Genomic structure of the zebrafish rho gene, with wild-type and rho mutant allele sequences shown below. The premature stop codon resulting from the frameshift mutation is indicated. (B) Whole-mount in situ hybridization illustrating rho expression in 5 dpf wild-type and rho mutant larvae. The position of primers used for probe synthesis is indicated in panel (A). (C) Confocal images showing the morphology of rod cell bodies (green) and OS (magenta) in the retinae of 5 dpf wild-type and rho mutant larvae. Rod cell bodies were labeled with the Tg(xops:GFP) transgene, while OS were labeled with the Tg(xops:mCherry-CT44) transgene. (D) Confocal images illustrating photoreceptor OS distribution and morphology in adult wild-type and rho mutant retinas. Rod cell bodies were labeled with Tg(xops:GFP), and OS were visualized with WGA staining. Rod OS are absent in rho mutants (stars). (E) Confocal images illustrating the morphology of rod OS in 7 dpf zebrafish with different transgenic backgrounds, as indicated. Rod OS were visualized with Tg(xops:mCherry-CT44) (red). (F) Quantitative analysis of the number of rod OS per section in 7 dpf zebrafish larvae. (G) Ectopic opsin expression in rod cells of adult rho mutants. Rod OS were visualized with Tg(xops:mCherry-CT44). Compared to the wild-type control and rho mutants, rhodopsin expression rescued rod OS morphology, whereas ectopic red opsin expression can partially rescue rods and induced a cone-like OS morphology. Enlarged views of boxed regions are shown below. (H–J) Quantification of rod OS morphology under different genetic backgrounds. (K) Statistical analysis of the distance from the base of the rod OS to the OPL, as shown in panels (G). DAPI (blue) marks cell nuclei. Scale bars: 200 μm in (B); 20 μm in (C, D, E, G). Data information: In (F), each dot represents the relative number of rod OS from the section of a larvae. Sample sizes per group are as follows: N(wt) = 16, N(rho) = 20, N(+Rho) = 12, N(+Lws1) = 12. S [file pbio.3003654.s004.tif]

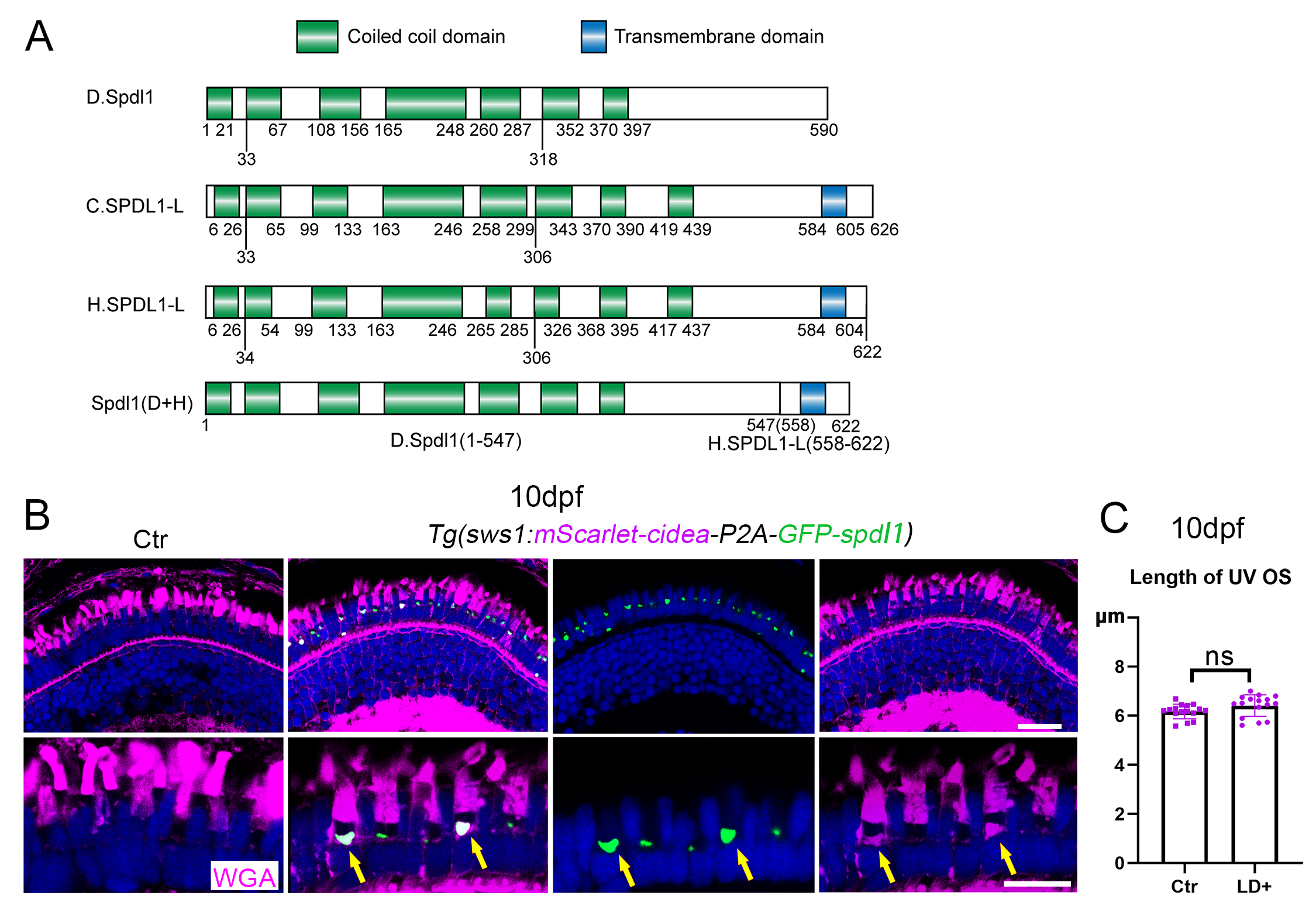

Supplement: S5 Fig — (A) Schematic representation of the domain structures of SPDL1 in zebrafish (D.Spdl1), chicken (C.SPDL1-L), and human (H.SPDL1-L). The chimeric protein used to induce lipid droplet formation contains the N-terminal of zebrafish Spdl1 (1–547) plus the transmembrane domain of human SPDL1 (558–622). Domain predictions were performed using InterPro. (B) Confocal images showing ectopic lipid droplet expression in UV cones of 10 dpf zebrafish larvae. The split green and magenta channels were shown on the right with arrows indicate ectopic LDs in the cell body of UV cones. (C) Quantification of UV cone OS length in 10 dpf larvae zebrafish. DAPI (blue) marks cell nuclei. Scale bar: 10 μm in (B). Data information: In (C), each dot represents one photoreceptor OS. Sample sizes per group are as follows: OS n(Ctr) = 16, n(LD+) = 16. Data for the wt and LD+ transgenic groups were derived from N = 5 and N = 8 zebrafish, respectively. Statistical significance was determined by the Mann-Whitney test. ns, not significant. The data underlying this Figure can be found in S1 Data. (TIF) [file pbio.3003654.s005.tif]

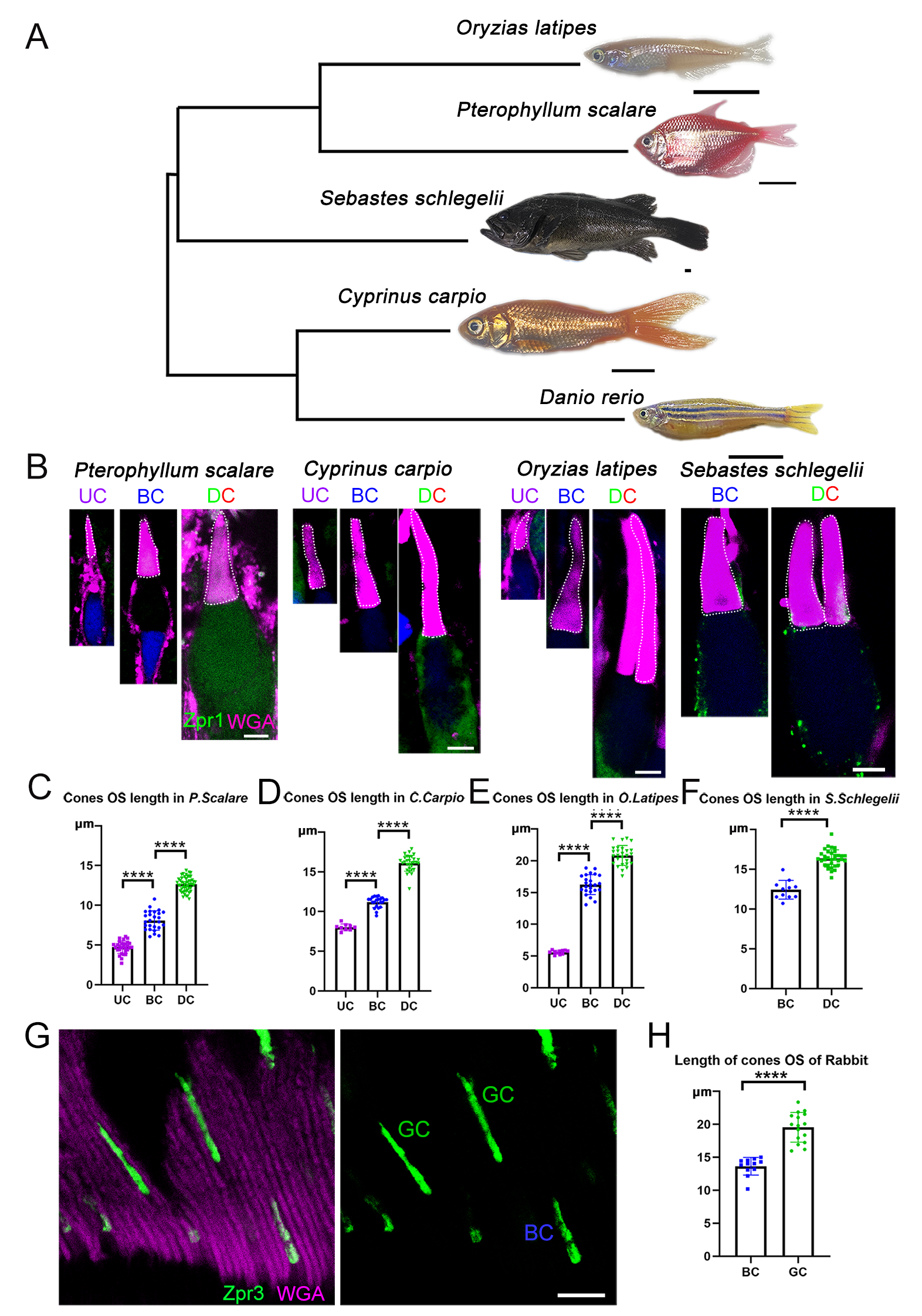

Supplement: S6 Fig — (A) Phylogenetic tree depicting the evolutionary relationships among Pterophyllum scalare, Cyprinus carpio, Oryzias latipes, Sebastes schlegelii, and Danio rerio. (B) Confocal images illustrating the morphology of cone OS in different species of teleost fish. The photoreceptor OS were labeled with WGA (red), and double cones were labeled with Zpr1 (green). Nuclei were stained with DAPI (blue). (C–F) Statistical analysis of cone OS lengths in various teleost fish species. (G) Confocal images illustrating the distribution and morphology of photoreceptor OS in adult rabbits. WGA (red) labels rod cell OS, while Zpr3 (green) can label the OS of the two types of cone cells. (H) Statistical analysis of cone cell OS lengths in rabbits. Scale bar: 5 μm in (B);10 μm in (H). Data information: In (C), each dot represents one photoreceptor OS. Sample sizes per group are as follows: OS n(UC) = 30, n(BC) = 26, n(DC) = 51. Data were derived from N = 3 Pterophyllum scalare per group. Statistical significance was determined by one-way ANOVA with Bonferroni’s post hoc test. In (D), each dot represents one photoreceptor OS. Sample sizes per group are as follows: OS n(UC) = 9, n(BC) = 22, n(DC) = 30. Data were derived from N = 3 Cyprinus carpio per group. Statistical significance was determined by one-way ANOVA with Bonferroni’s post hoc test. In (E), each dot represents one photoreceptor OS. Sample sizes per group are as follows: OS n(UC) = 11, n(BC) = 23, n(DC) = 27. Data were derived from N = 3 Oryzias latipes per group. Statistical significance was determined by one-way ANOVA with Bonferroni’s post hoc test. In (F), each dot represents one photoreceptor OS. Sample sizes per group are as follows: OS n(BC) = 33, n(DC) = 23. Data were derived from N = 3 Sebastes schlegelii per group. Statistical significance was determined by the Student t test. In (H), each dot represents one photoreceptor OS. Sample sizes per group are as follows: OS n(BC) = 12, n(GC) = 16. Data were derived from N = [file pbio.3003654.s006.tif]

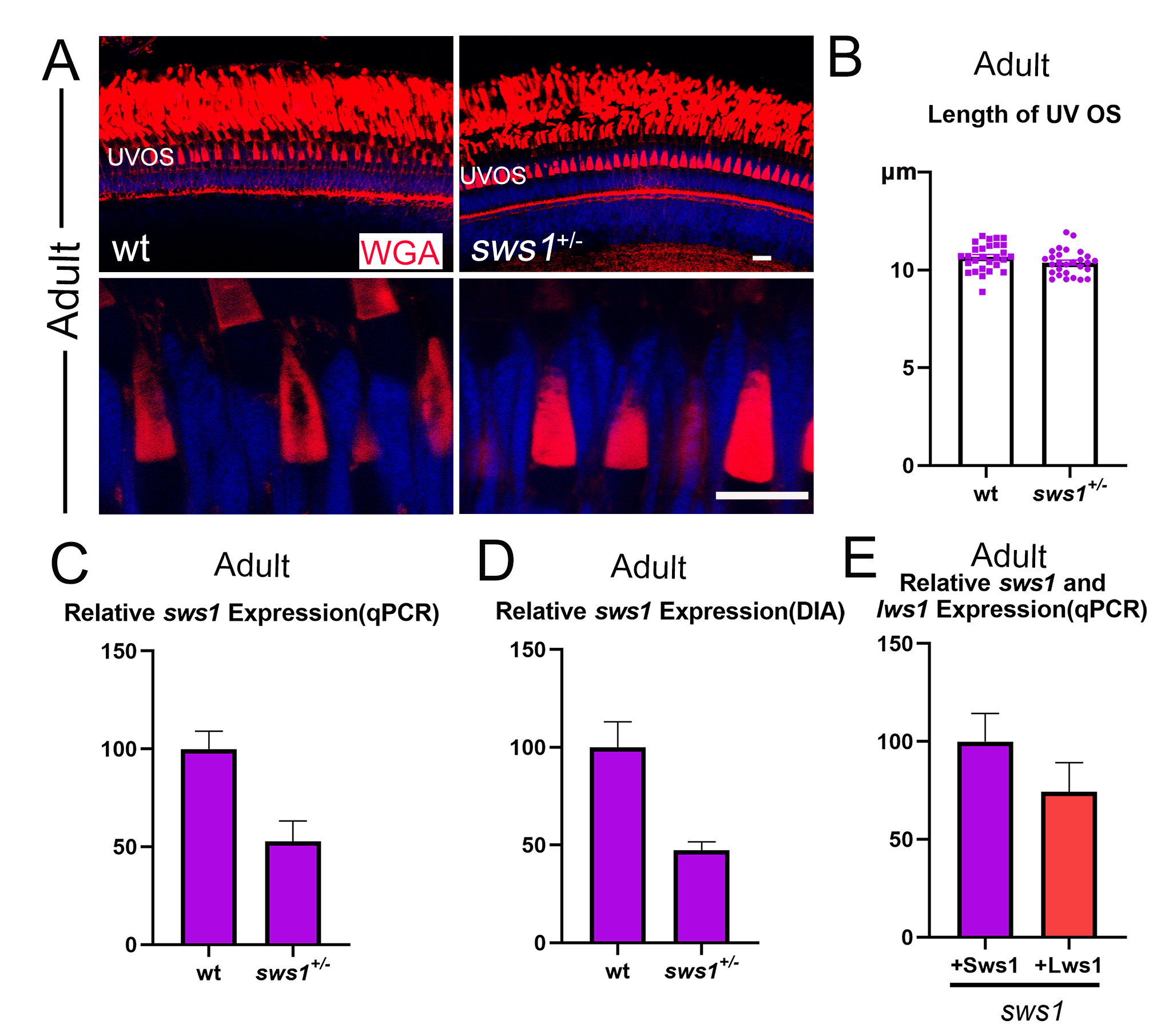

Supplement: S7 Fig — (A) Confocal images of UV cone OS in wild-type and sws1⁺/− heterozygous mutant zebrafish. Nuclei are stained with DAPI (blue). (B) Quantification of UV cone OS length in wild-type and sws1⁺/− zebrafish. (C) qPCR of relative sws1 mRNA expression levels in wild-type and sws1⁺/− zebrafish. (D) DIA of relative sws1 protein expression levels in wild-type and sws1⁺/− zebrafish. (E) Relative mRNA expression of sws1 and lws1 in transgenic rescue lines. Scale bars: 15 μm (low-magnification) and 10 μm (high-magnification) in (A). Data information: In (B), each dot represents one photoreceptor OS. Sample sizes per group are as follows: OS n(wt) = 28, n(sws1⁺/−) = 28. Data were derived from N = 5 zebrafish per group. Statistical significance was determined by the Student t test. In (C, E), the qPCR for each sample was performed in technical triplicates. ns, not significant. The data underlying this Figure can be found in S1 Data. (TIF) [file pbio.3003654.s007.tif]

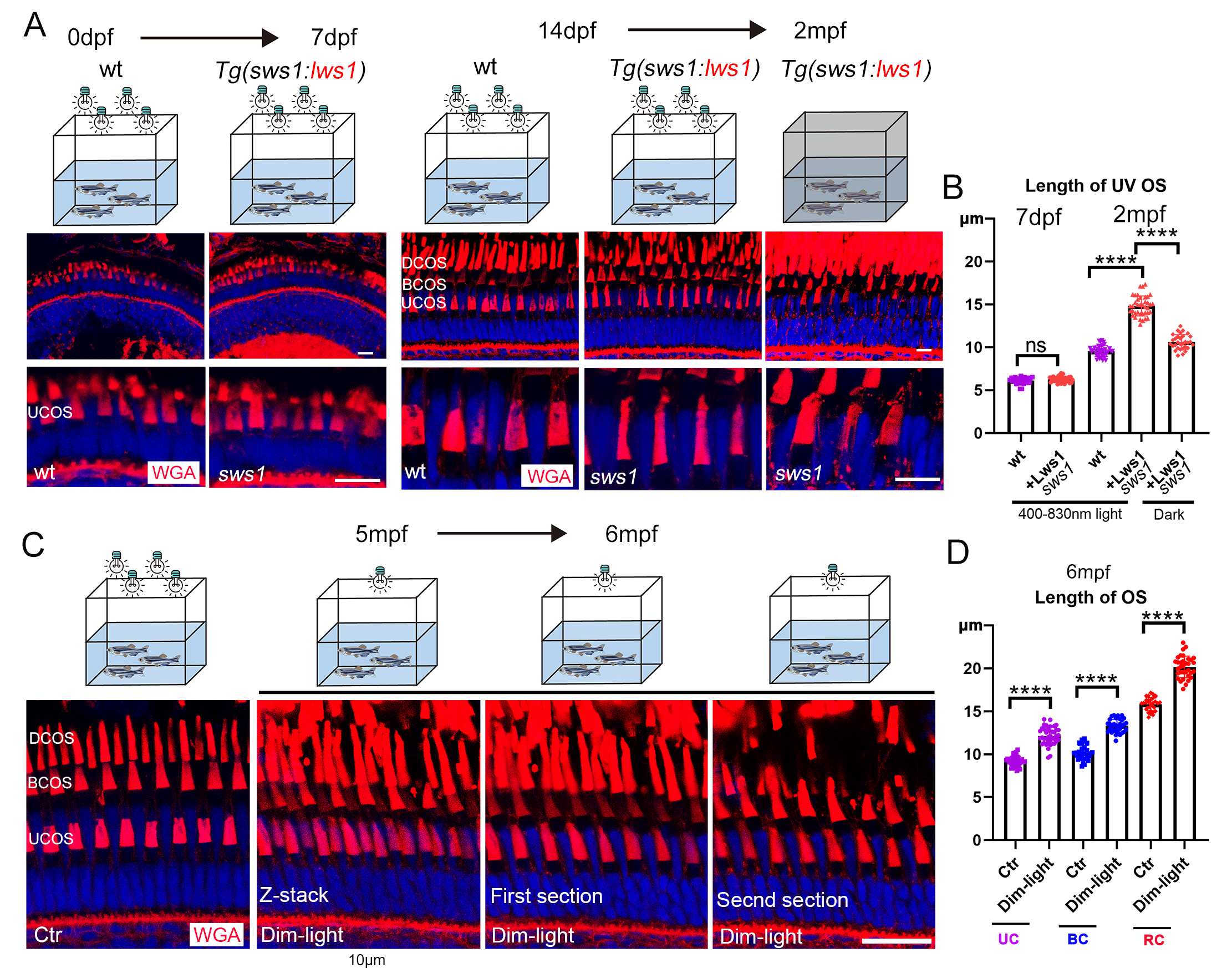

Supplement: S8 Fig — (A) Confocal images showing the morphology of cone OS in wild-type or sws1 mutants carrying Tg(sws1:lws1) transgene. The schematic diagram of the strategy of the light/dark treatment is shown on top of each figure. Enlarged views of the UV cone OS is shown on the bottom. OS were labeled with WGA. (B) Quantification of UV cone OS lengths under normal light and dark conditions with different genetic background as indicated. (C) Confocal images showing the lengths of cone OS following one month of exposure to normal and dim light starting from 5mpf old adult zebrafish. Schematic diagram of low-light intensity treatments was shown on the top. OS were labeled with WGA. To distinguish the length of blue and double cone OS, different focus planes were shown on the right. Z-stack image shows the maximum intensity projection image. (D) Quantitative analysis of cone OS lengths under different light intensities. DAPI (blue) marks cell nuclei. Scale bars: 10 μm in (A, C). Data information: In panel (B), each dot represents one photoreceptor OS. Sample sizes per group are as follows: OS n(wt 7dpf) = 26, n(+Lws1 7dpf) = 30, n(wt 2mpf) = 40, n(+Lws1 2mpf light) = 39, n(+Lws1 2mpf Dark) = 31. Data were derived from N = 6 zebrafish per group. Statistical significance was determined by one-way ANOVA with Bonferroni’s post hoc test. In (D), each dot represents one photoreceptor OS. Sample sizes per group are as follows: OS n(Ctr UC) = 36, n(Dim-light UC) = 40, n(Ctr BC) = 29, n(Dim-light BC) = 32, n(Ctr RC) = 31, n(Dim-light RC) = 34. Data were derived from N = 5 zebrafish per group. Statistical significance was determined by one-way ANOVA with Bonferroni’s post hoc test. **** p < 0.0001; ns, not significant. The data underlying this Figure can be found in S1 Data. (TIF) [file pbio.3003654.s008.tif]

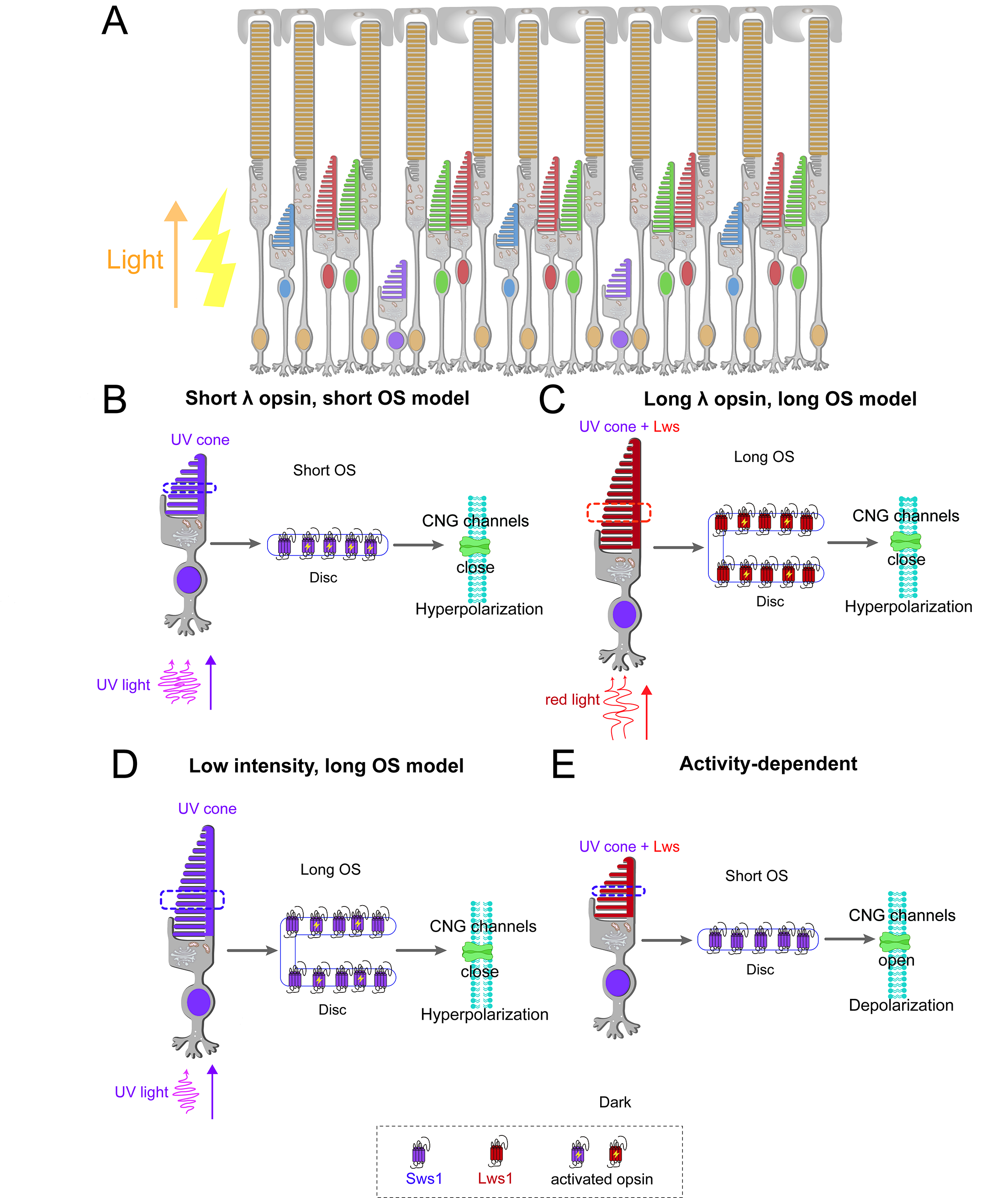

Supplement: S9 Fig — (A) Schematic illustrating the path of light through the layered zebrafish retina. The different arrangement of rod and cone OSs in the photoreceptor layer were shown. (B–E) Schematic diagrams illustrating a model for how cone photoreceptor OS length is regulated. For effective phototransduction, the outer segment must generate sufficient membrane potential through cyclic nucleotide-gated (CNG) channels (right). Closure of these channels may require a minimum number of activated opsin molecules. For example, at least four activated opsins (yellow) may be needed to induce adequate CNG channel closure. Because short-wavelength ultraviolet (UV) light has higher photon energy, it can meet this activation threshold with only a single opsin layer (B). In contrast, long-wavelength red light, which activates red opsins less efficiently, requires additional opsin layers to capture enough photons to reach the threshold (C). Likewise, reduced light intensity—whether caused by lipid droplets or low-light environmental conditions—decreases the likelihood of photon capture, potentially necessitating additional opsin layers along the light path to maintain sufficient activation (D). Finally, in the absence of light, such elongation of the OS may not occur even when long-wavelength red opsins are expressed (E). (TIF) [file pbio.3003654.s009.tif]
