# Supplementary material for: Light intensity and opsin sensitivity shape the morphology of cone photoreceptor outer segments
Source: PLoS Biol. 2026 Feb 18;24(2):e3003654. doi: 10.1371/journal.pbio.3003654 (PMC12915902; doi:10.1371/journal.pbio.3003654)
Supplement: S1 Table — (DOCX) [file pbio.3003654.s010.docx]

**S1 Table: Transgenic Zebrafish Generated in this work**

| **Transgenic zebrafish** | **Functions** |
| --- | --- |
| 1. *Tg(sws1:HA-tdTomato-CT44)* | Labels UV cones OS. |
| 2 *Tg(sws2:HA-tdTomato-CT44)* | Labels blue cones OS. |
| 3. *Tg(Hsp70l:GFP-CT44)* | Labels all cones OS and simulates the transport of rod and cone opsins. |
| 4. *Tg(xops:GFP)* | Labels the cell bodies of rods. |
| 5. *Tg(sws1:sws1)* | Expresses exogenous Sws1 in UV cones. |
| 6. *Tg(sws1:mws3)* | Ectopically expresses Mws3 in UV cones. |
| 7. *Tg(sws1:lws1)* | Ectopically expresses Lws1 in UV cones. |
| 8. *Tg(sws1:turtle LWS)* | Ectopically expresses LWS in UV cones. |
| 9. *Tg(sws1:rho)* | Ectopically expresses Rho in UV cones. |
| 10. *Tg(sws1:GPR14)* | Ectopically expresses GPR14 in UV cones. |
| 11. *Tg(sws1:HA-sws1)* | Expresses HA-Sws1 in UV cones. |
| 12. *Tg(xops:lws1)* | Ectopically expresses Lws1 in rods. |
| 13. *Tg(xops:rho)* | Ectopically expresses Rho in rods. |
| 14. *Tg(sws1:GFP)* | Labels the cell bodies of UV cones. |
| 15. *Tg(sws1:sws1-GFP)* | Labels UV cones OS. |
| 16. *Tg(xops:mCherry-CT44)* | Labels the OS of rods. |
| 17. *Tg(sws1:mScarlet-cidea-P2A-GFP-spdl1)* | Ectopically expresses lipid droplets in UV cones. |
