# Supplementary material for: Light intensity and opsin sensitivity shape the morphology of cone photoreceptor outer segments
Source: PLoS Biol. 2026 Feb 18;24(2):e3003654. doi: 10.1371/journal.pbio.3003654 (PMC12915902; doi:10.1371/journal.pbio.3003654)
Supplement: S2 Table — (DOCX) [file pbio.3003654.s011.docx]

**S2 Table: Primer Sequences**

| **Primers for Gateway BP reaction** | |
| --- | --- |
| *sws1*(without termination codon)-F | GGGGACAAGTTTGTACAAAAAAGCAGGCTCCACCATGGACGCGTGGGCCGTTCAATTC |
| *sws1*(without termination codon)-R | GGGGACCACTTTGTACAAGAAAGCTGGGTATGCAGACACAGATGAGGTTTCG |
| *sws1*-F | GGGGACAAGTTTGTACAAAAAAGCAGGCTCCACCATGGACGCGTGGGCCGTTCAATTC |
| *sws1*-R | GGGGACCACTTTGTACAAGAAAGCTGGGTATTATGCAGACACAGATGAGGTTTCG |
| *mws3-F* | GGGGACAAGTTTGTACAAAAAAGCAGGCTCCACCATGAACGGGACAGAAGGGAGCA |
| *mws3-R* | GGGGACCACTTTGTACAAGAAAGCTGGGTACTATGCAGGAGACACAGAGGAC |
| *lws1-F* | GGGGACAAGTTTGTACAAAAAAGCAGGCTCCACCATGGCAGAGCATTGGGGAGATG |
| *lws1-R* | GGGGACCACTTTGTACAAGAAAGCTGGGTATTATGCAGGAGCCACAGAAGAC |
| *rho-F* | GGGGACAAGTTTGTACAAAAAAGCAGGCTCCACCATGAACGGTACAGAGGGACC |
| *rho-R* | GGGGACCACTTTGTACAAGAAAGCTGGGTATTACGCCGGAGACACGGAGC |
| *GPR14-F* | GGGGACAAGTTTGTACAAAAAAGCAGGCTCCACCATGGCACTTACACCTGAATC |
| *GPR14-R* | GGGGACCACTTTGTACAAGAAAGCTGGGTATCAAGCGGGTGCACGGGGCCCTT |
| *HA-sws1-F* | GGGGACAAGTTTGTACAAAAAAGCAGGCTCCACCATGTACCCATACGATGTTCCAGATTACGCTATGGACGCGTGGGCCGTTCAATTC |
| *HA-sws1-R* | GGGGACCACTTTGTACAAGAAAGCTGGGTATTATGCAGACACAGATGAGGTTTCG |
| **Primers for Infusion** | |
| vector-F | TACCCAGCTTTCTTGTACAAAGTGG |
| vector-R | GCCTGCTTTTTTGTACAAACTTG |
| mScarlet-*cidea*-F | GTTTGTACAAAAAAGCAGGCTGCCACCATGGTGAGCAAGG |
| mScarlet-*cidea*-R | CGCTTGAGCATTCCTCATCTCCG |
| P2A-GFP-F | AGATGAGGAATGCTCAAGCGCCACGAACTTCTCTCTGTTAAAGC |
| P2A-GFP-R | GGATCCTCCCTTGTACAGCTCGTCCATGCC |
| *spdl1(D.+H.)*-F | AGCTGTACAAGGGAGGATCCGGTGGATCTGG |
| *spdl1(D.+H.)*-R | TTGTACAAGAAAGCTGGGTATCAAGAGTCCTTTCCCTTTGCA |
| **Primers for Probe** | |
| *sws1*-F | CAGCCTCTCAACTACATCTT |
| *sws1*-R | ACCATTACAACAACCATCCT |
| *rho*-F | GGCTATGGTCGCTTGTAG |
| *rho*-R | GTGACGGAACTGCTTGTT |
| **Primers for QPCR** | |
| *sws1*-F | GATGGTCCTTGGCTGTTC |
| *sws1*-R | TTCCTTCACCTGCTCCAT |
| *lws1*-F(The forward primer was designed within the 5' UTR region of the sws1 promoter to avoid amplifying the endogenous *lws1* gene.) | CTTGTTGACTCCACCAGGAC |
| *lws1*-R | CTCAAAGGGATCCTTGGTGT |
| *βactin*-F | CCGTGACATCAAGGAGAAGC |
| *βactin*-R | TACCGCAAGATTCCATACCC |
