# Supplementary material for: Light intensity and opsin sensitivity shape the morphology of cone photoreceptor outer segments
Source: PLoS Biol. 2026 Feb 18;24(2):e3003654. doi: 10.1371/journal.pbio.3003654 (PMC12915902; doi:10.1371/journal.pbio.3003654)
Supplement: Attachment — Submitted filename: PBIOLOGY-D-25-02009 Response.pdf [file pbio.3003654.s013.pdf]

REVIEWS:

Reviewer #1 (Juan Angueyra signed his report): SUMMARY OF REVIEW:

This paper aims to highlight the role of visual opsins in photoreceptor outer segment (OS) morphology, with the goal of providing insight to outer segment development and plasticity. Through a series of novel experiments in both larval and adult zebrafish, using new mutant and transgenic lines, the authors demonstrate that OS shape is correlated with opsin expression, opsin sensitivity, and light intensity. In addition, the authors carry additional manipulations (hormonal and environmental) and cross species comparisons known to create differences in opsin expression and find corresponding changes in OS shape.

These results are exciting and interesting.

Finally, the authors propose a model on how photoreceptor OS is regulated by light energy. We believe that some changes in rephrasing this claim, and acknowledging the possibility of other mechanisms would improve the overall impact and readability, especially for non-retina experts. We also suggest the authors refine the figure legends and Methods to ensure the reproducibility.

What are the main claims of the paper and how significant are they for the discipline? Are these claims novel?

Claim 1: Specific opsin expression drives OS morphology.

Novel = Yes. Although the influence of opsin levels on outer segment length is well described, this study includes clever ectopic expression of opsins, which validates a mechanism where specific properties of each opsin control OS morphology

Claim 2: Correlation between OS length and wavelength sensitivity of opsin expressed

Novel = Yes. Cell-specific manipulations added novelty to well-described literature.

Claim 3: Control of OS shape is activity dependent

This mechanism is not well supported by the presented data.

Is this paper outstanding in its discipline?

Yes. This study uses novel and insightful approaches, presents interesting findings, and opens multiple possibilities for follow up studies. In particular this will lead to experiments to identify mechanisms able to control cell shape, which is relevant to many fields.

Who would find this paper of interest? Why?

This could be of general interest to cell biologists, retinal biologists, and visual neuroscientists. Researchers interested in understanding the mechanisms that govern morphology differences across

cell subtypes, particularly in the retina, will find this particularly interesting because it may provide a new bridge between structure and function in neurons.

Are the claims properly placed in the context of the previous literature? Have the authors treated the literature fairly?
Yes, the authors cite relevant literature to their field and everything is discussed in context to previous literature.

Do the data and analyses fully support the claims? If not, what other evidence is required?
Claim 1: Specific opsin expression drives OS morphology.
Supporting evidence: Yes; ectopic, environmental, and hormonal manipulations. In addition, cross species comparisons. All lines of evidence reach similar conclusions.
Claim 2: Correlation between OS length and wavelength sensitivity of opsin expressed
Supporting evidence: Yes.
Claim 3: Control of OS shape is activity dependent
Supporting evidence: No, the evidence provided does not uniquely support this claim. We recomm end the authors to modify the claim as it stands.
Further Evidence Required:
To support this claim, a manipulation that modifies activity is required. For example, mutations in phototransduction proteins that cause blindness could be a first step towards this goal.

Answer:
Thank you for these constructive comments and suggestions. We agree that confirming these results in phototransduction mutants would further strengthen our conclusions. However, generating such mutants with both the mutant and transgenic backgrounds required more than six months, making this approach impractical within the revision timeframe. In addition, zebrafish phototransduction mutants (such as cone-specific *pde6* genes) may undergo rapid cone degeneration (PMID: 18077698), which makes it difficult to test our hypothesis in these backgrounds.

To further support the role of neural activity in regulating cone morphology, we have performed additional experiments showing that the morphological changes of cone outer segments are significantly reduced when fish are raised in darkness, a condition

expected to minimize neural activity. Although we cannot fully exclude contributions from other factors, these results suggest that neural activity plays an important role in the regulation of cone outer segment morphology. We have included these new data in the revised manuscript (Fig. S8).

Other possible (nonactivity dependent) mechanisms need to be excluded. For example, OS morphology could be dictated by sequence differences in opsins, differences in ciliary transport of the misexpressed opsins, or changes rates of disc shedding by the RPE, amongst others.

Answer:

Thanks for these valuable suggestions. We agree that we cannot exclude this possibility. We have now included a discussion of potential factors that may influence outer segment morphology in the revised manuscript.

Would additional work improve the paper? How much better would the paper be if this work were performed and how difficult would it be to do this work?

We recommend revisions of the text, without additional experiments.

If the paper is considered unsuitable for publication in its present form, does the study itself show sufficient potential that the authors should be encouraged to resubmit a revised version?

The authors should resubmit a revised version after thorough revision of the methods section to make the manuscript suitable for publications (see below). In addition, the authors should revise or soften their claim regarding that the mechanism that underlies OS length is activity dependent as the data is suggestive but insufficient to exclude other possible mechanisms.

Are original data deposited in appropriate repositories and accession/version numbers provided for genes, proteins, mutants, diseases, etc.?

N/A

Is the manuscript well organized and written clearly enough to be accessible to non-specialists?

Yes.

Are details of the methodology sufficient to allow the experiments to be reproduced?

No.

Imaging: not enough details provided. We recommend that the authors follow established standards in the field for reporting methods for fluorescent and electron-microscopy images (e.g. https://doi.org/10.1038/s41592-021-01156-w)

qPCR: key steps of protocol, experiments and analysis have been omitted. For example, no primers were provided for the normalization gene, no mention of RNA extraction protocol, of quality checks on cDNA, etc. (e.g. https://www.biorxiv.org/content/10.1101/2024.12.04.626769v1.full.pdf)

Light exposure experiments: no spectral data provided for LEDs used. Given the claims about spectrum and activity-dependent mechanisms, this information is highly relevant.

Statistics: none of the experiments have explicit mention of biological replicates, and relevant details to assess confidence on statistical analysis are omitted, including details (other than p-value) of statistical tests (e.g. https://pmc.ncbi.nlm.nih.gov/articles/PMC6639881/; https://plos.org/resource/how-to-report-statistics/)

Animals: While the results mention specific days post-fertilization, the manuscript lacks clarity regarding which developmental time points were used for comparative analysis with teleost species. Given that there is both larval and adult data presented, explicit mention of age is required for all datasets.

We recommend a thorough revision of the methods section. This should also include revision of figure legends; in particular "Quantitative analysis of …" should be replaced with all the details about statistical tests (n, degrees of freedom, value of t-test, etc.). There is also no description of what each dot in bar plots represents. If each dot represents a single OS, it is required to report how many images were analyzed for each experiment and if the images correspond to different animals or not (which correspond to true biological replicates), to be able to assess if this study has enough statistical power.

Answer:

We appreciate these detailed suggestions. In the revised manuscript, we have thoroughly revised the Methods section and figure legends to address all the points raised. We have also included all necessary statistical information within each figure panel to improve clarity and reproducibility. Moreover, we have attached an Excel file including all the numerical values that were used to generate statistical graphs.

Regarding our conclusion that OS length is activity-dependent, we agree that the current data are suggestive but do not fully exclude other possible mechanisms. We

have therefore revised the text to soften this claim and now more cautiously interpret our findings within the context of alternative explanations. These revisions are incorporated throughout the Results and Discussion sections.

PLOS Biology encourages authors to publish detailed protocols and algorithms as supporting infor mation online. Do any particular methods used in the manuscript warrant such treatment?

No.

Other Minor Edits:

Many images are not color-blind friendly. We recommend using magenta instead of red when red-green distinctions are important

Answer:

In the revised version, we have replaced red with magenta in all figures where red–green distinctions are important.

Much of the text on the fluorescent images is difficult to discern. For example, the red WGA text can be difficult to read where it overlays the images, and would benefit from improved contrast or alternative labeling approaches.

Unclear if scale bar is consistent, and this is sometimes not mentioned. For example, in Figure 2 the dimensions for the scale bar is not mentioned for F-I even though there is a scale bar for I shown.

Having a key for colors in box plots could be helpful, and could be kept consistent for similar para meters in other plots.

Properly report the replicate size and the sample size.

Answer:

We have carefully checked all figures and revised labels and descriptions to ensure that they are clear, accurate, and easy to interpret.

Figure 2A; does the 10 bp insertion create a premature STOP codon?

Answer:

Yes, this mutation introduces a premature stop codon due to frameshift, resulting in a truncated UV opsin protein. We have revised the figures in the updated manuscript.

Figure 3: Are both WGA and cidea labeled red? Is Cidea expressed in the transgenic cones?

Answer:

Yes, both WGA and cidea (mScarlet-tagged) were displayed in red. However, they can be distinguished by their distinct localization patterns: WGA staining does not label lipid droplets, while the red fluorescence from transgenic Cidea expression is predominantly localized in the inner segment. The mScarlet-Cidea fusion protein colocalizes with GFP-Spdl1 in lipid droplets; however, the strong GFP-Spdl1 signal partially obscured the mScarlet-Cidea signal. In the revised version, we have separated these two channels to more clearly illustrate their localization (Fig 4 and Fig S5).

Figure S3A: Why is there no quantification with GFP14? Why 5 dpf for only this experiment?

Answer:

Because neither GPR14 nor the tdTomato-tagged C-terminal fragment of rhodopsin (note: this fragment contains only the last transmembrane domain, not the full-length protein) was able to rescue the UV opsin mutants, UV-cone outer segments were completely absent in these mutants even at the larval stage. For this reason, we presented only the larval mutant retinae in the original submission. Since UV cones were fully degenerated in mutants carrying these transgenes, we initially did not include quantitative analyses. Given that this manuscript is a short report, and these experiments are not essential to the main conclusions, we have removed these data in the revised version due to space limitations, as suggested by other reviewers.

Figure S3G: Why is there no quantification? Is the rescue full? The example images seem to suggest that there are more UV cones in the rescue than in WT?

Answer:

Yes, *Tg(sws1:sws1)* can fully rescue the UV cone defects observed in the UV opsin mutants. We have included the corresponding statistical analyses in the revised version of the manuscript (Fig S2).

Figure S4B: What is relative #? How is this calculated and why is it not absolute?

Answer:

To compare the number of UV-cone outer segments across different transgenic backgrounds, we quantified UV-cone outer segments by a fix length of the photoreceptor layer. This value is presented as the number per arbitrary unit. These methodological details have now been added to the methods and figure legends in the revised manuscript.

Page 13: "Similar to humans, zebrafish cones are classified into S-cones (blue), M-cones (green), and L-cones (red) (Perkins & Fadool, 2010). Additionally, zebrafish feature a specialized type of cone that is sensitive to UV light" is misleading since the human S-cone is actually related to the zebrafish UV cone and not the Scone (see Baden et al, 2025: https://doi.org/10.1371/journal.pbio.3003157)

Answer:

We apologize for the confusion. In the revised manuscript, we have updated the terminology according to the nomenclature recommended by the reviewer.

Include the spectrum of LED panel used for light exposure experiment

Answer:

We have mentioned the spectrum of the LED lights in the revised text.

"Reducing photon energy (e.g., switching to longer wavelengths) or decreasing photon capture (e.g., via additional lipid droplets or in low-light environments) lowers the likelihood of photopigment activation": this is not true. For example, according to the spectral sensitivity curves for L-opsins, switching from blue light to red light (high energy to low energy) increases photopigment activation. Photopigment activation depends on the intensity AND spectrum of the stimulating light AND the spectral sensitivity of the opsin.

Answer:

Thank you for pointing out this issue. We agree with the reviewer that switching from blue to red light (high to low photon energy) can indeed increase photopigment activation for L-opsins, because this shift moves from a region of lower sensitivity to the peak of the opsin's spectral sensitivity curve. Photopigment activation depends on the interaction between light intensity, spectrum, and the spectral sensitivity of the opsin.

In our experiments, however, we manipulated the expression of different opsins within the same cone type, and each ectopically expressed opsin was stimulated near its own peak sensitivity. Under these conditions, we intended to emphasize that, for a given opsin at its optimal wavelength, higher-energy photons generally have a higher probability of causing isomerization than lower-energy photons at their respective optimal wavelengths. We have now rewritten this paragraph in the revised manuscript and clarified the conceptual model to avoid misunderstanding.

Suggestions:

Hebbian plasticity: The classic Hebb's rule is about the connection between neurons, and although famous, not the only example of activity dependence. We find it challenging to understand the direct comparison between Hebbian plasticity between two cells and the changes in cell shape presented in this manuscript.

Answer:

Thank you for this valuable comment. We agree that classical Hebbian plasticity, which describes activity-dependent changes in synaptic connections between neurons, is not an appropriate example for comparison with the activity-dependent changes in cone cell morphology described in our study. We have revised this section accordingly and removed the direct reference to Hebbian plasticity in the updated manuscript.

Restructure the discussion to match flow of the introduction:The authors discuss environmental changes as a factor for altering OS shape. We recommend that the authors move the environmental changes section before discussion about oil droplets. The reviewers believe this structure will benefit the flow of the manuscript. If not, alter the introduction to highlight cellular modifications to improve/alter/enhance particular functions (such as oil droplets in photoreceptors).

Answer:

Thank you for this helpful suggestion. We agree that reorganizing these sections will improve the logical flow of the manuscript. In the revised version, we have reordered this parts by moving the section on environmental influences to precede the section on oil droplets.

Clarify times of experiments and adult vs larval data: The authors provide insightful data from both adult and larval stages of zebrafish, but it becomes unclear which pieces of data are being referred to. For example it is explained that the five photoreceptor subtypes are easily distinguished using wheat germ agglutinin (WGA) staining, but it is unclear at what developmental timepoint this is at. We suggest the authors add age to every figure once and distinguish when different ages are used. This will make the results explicitly clear when the authors are referring to morphology in adult vs. larval/developmental stages.

Answer:

We appreciate the reviewer's suggestion. In the revised manuscript, we have now clearly indicated the developmental stage—larval or adult—for each experiment in the corresponding figures and figure legends to avoid any ambiguity.

Suggestion for the discussion: the authors state that OS morphology (mainly length, width, and shape) is directly related to the opsin that is expressed. But does not acknowledge scenarios where opsins are co-expressed in the same cells (mice M/S gradient, cichlid/salmon UV/S transition). Do the authors think that OS morphology changes in hybridized cell identities?

Answer:

Thank you for raising this important point. At present, we do not have sufficient data to compare outer segment morphology between single-opsin and hybridized cone types in systems such as mouse M/S cones or the UV/S transitional cones in salmonids. Based on our model, we speculate that the morphology of hybrid cones may be more similar to that of cones expressing the shorter-wavelength–sensitive opsin. For example, in our system, the outer segment morphology of UV cones ectopically expressing LWS opsin resembles that of native UV cones, despite simultaneously expressing both endogenous UV and ectopic LWS opsins (see below). However, additional experimental evidence will be required to fully address this question.

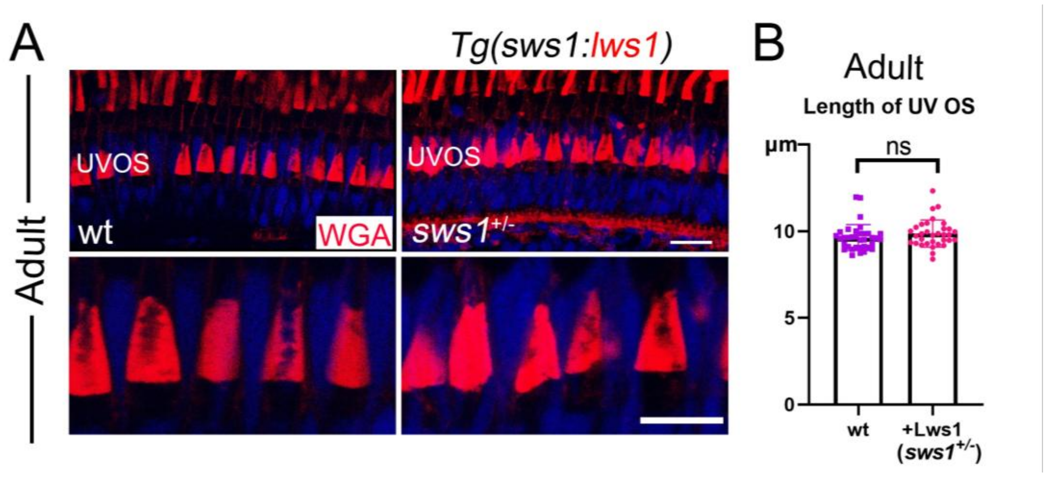

Suggestion for the discussion: The term morphology encompasses quite a bit when looking at photoreceptor cell structure: in addition to the outer segment, did the authors notice any changes in the rest of the cell morphology? (inner segment, cell bodies, ribbon synapses).

Answer:

Thank you for this comment. In our manuscript, "morphology" specifically refers to the photoreceptor outer segment. We have also examined other cellular compartments

and observed inherent differences among photoreceptor types—for example, rod photoreceptors have elongated fibers connecting the inner segment to the cell body, while UV cones have shorter connecting fibers. Nuclear shapes also vary: double cone nuclei are rod-shaped, whereas other photoreceptor nuclei are generally elliptical. Importantly, in our experiments with ectopic opsin expression, we did not observe significant changes in the inner segment and cell body between control and experiment groups (see below).

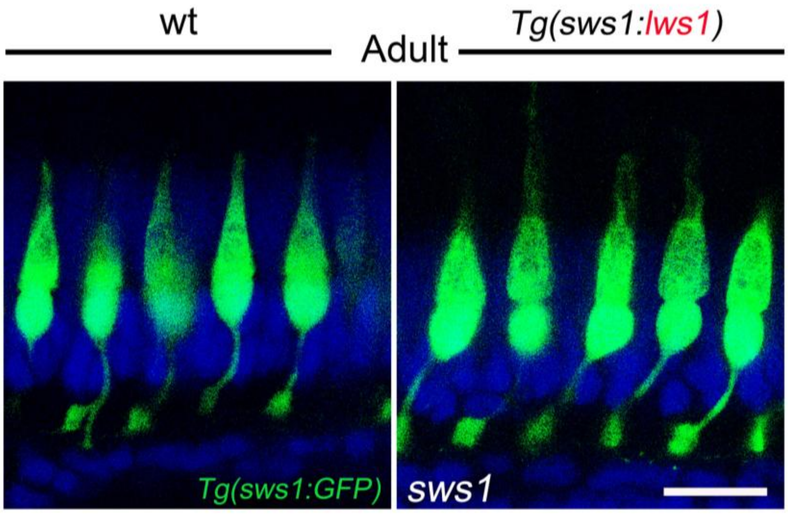

Reviewer #2: Photoreceptors have outer segment (OS) regions specialized to absorb photons and create a visual response. The manuscript by Xu et al presents a set of data to support an interesting hypothesis, that cone photoreceptor OS size and shape is directly related to light sensitivity, and is adaptable depending on the conditions. Using zebrafish as a model, the authors characterize the OS dimensions of the four cone subtypes, demonstrating that photoreceptors detecting longer wavelengths have longer and wider OSs. The replacement of UV opsin in the short cones with another cone opsin was sufficient to convert the OS morphology to match the opsin. Surprisingly, rod opsin did not change the UV cone morphology. On the other hand, expression of red opsin in rods lacking rho created rods with OSs shaped similar to red cones and also shifted the OS position to a more inner layer. Expression of a long wavelength turtle opsin in sws1-deficient UV cones led to a tripling in OS length compared to wildtype UV cones. Treatment with thyroid hormone, which promotes the switch from Vitamin A1 to A2-based photopigments (A2 is red-shifted) promoted lengthening of red OSs. Another experiment involved the triggering of lipid droplet formation in the inner segment or growing fish in reduced light conditions, in both cases limiting light passage into the OS. The hypothesis was that the OS would lengthen to accommodate the reduction in light

input, which was indeed the finding. Finally, the authors examined a variety of fish species, observing similar relationships between cone height and wavelength. For black rockfish that lives at depth when wild but is cultivated in shallower waters, the cone photoreceptors were longer in the deep-dwelling population. Overall, the authors used multiple experimental approaches to support their hypothesis that cone OSs adapt to their light-detecting functions.

Generally, the paper is well written and organized. Some of the experiments are quite ingenious. The group uses a wide breadth of experiments to support their hypothesis. My comments are mostly about improvements for the experimental analysis.

Answer:

We sincerely appreciate the reviewers' positive assessment of our manuscript and their thoughtful comments.

Major comments:

1) The transgenic line sws1:sws1-GFP is used to mark OS. But the line is massively overexpressing sws1 opsin in these cells. Evidence is needed to show that morphology is unaffected in these cells.

Answer:

Thank you for this comment. We have measured and compared the outer segment length of UV cones between adult Tg(*sws1:sws1-GFP*) zebrafish and wild-type controls. Our results demonstrate that the OS lengths are comparable between these two groups (Fig S2F-G).

2) The WGA labelling is curious. WGA is typically used as a rod OS marker, so the specific labelling of cone OS is surprising. Can the authors explain or justify the discrepancy? Is there evidence in the literature for its use as a cone OS label, especially for UV cones.

Answer:

Thank you for this comment. WGA binds to specific glycans on the cell surface, and photoreceptor OS membranes are enriched in glycoproteins (including opsins) and glycolipids, providing abundant binding sites for WGA. Although WGA is frequently used as a rod OS marker, it has also been reported to label cone OS in multiple species. For example, in the cat retina, WGA strongly labels both rod and cone outer segments as well as the cone sheath (Fariss, Anderson et al., 1990). WGA has also been used as a general photoreceptor marker in avian retinae (Garlipp, Nowak et al., 2012).

In our experiments, we observed that WGA can label both rod and cone OS in teleosts, though with varying efficiencies. The signals were particularly strong in rods, double cones, and UV cones, whereas blue cones showed relatively weaker labeling. These differences likely reflect distinct glycoconjugate compositions among photoreceptor subtypes.

3) What are the data points shown in the graphs throughout the paper? Are they measurements for individual cells, section, eyes, or fish?

Answer:

Thank you for pointing out this issue. In the revised manuscript, we have added the information for each data points in the corresponding figure legends.

4) t-tests are the only statistics described, which is not appropriate for multiple groups compared to one wildtype group, unless with a Bonferroni correction.

Answer:

Thank you for this suggestion. In the revised manuscript, we have updated the statistical analyses accordingly. Specifically, for comparisons involving multiple groups, we replaced the Student's t-test with one-way ANOVA test followed by Bonferroni's correction for multiple comparisons or Kruskal-Wallis test followed by Dunn's correction for multiple comparisons. This change ensures that all multiple comparisons are evaluated using appropriate statistical methods.

5-6) If the UV cones with swapped opsins remain in the same layer, then TEM could be used to support the conclusion of altered morphology and would be a strong addition, particularly for seeing changes in width of the UV cone OS. The rho-/- retinas with lws1 expression must look vastly different from wt, and even the rho rescue looks disorganized. TEM would again benefit this characterization, and maybe also further immuno characterization of the retinal layering.

Answer:

Thank you for this excellent suggestion. In the revised manuscript, we have performed TEM analysis on UV cones expressing lws1. The TEM results confirm that these UV cones remain in the same retinal layer and display elongated outer segment morphology (Fig. 2 I-K), consistent with our immunofluorescence observations (Fig. 2 E).

For the *rho*$^{-/-}$ retinas expressing lws1, the rod photoreceptors with partially rescued OSs appear highly disorganized and are not well aligned within the photoreceptor layer. This disorganization makes it difficult to reliably identify and analyze these OS structures using TEM. Based on our current data, *lws1* expression does not fully restore normal rod OS morphology even at larvae stages (Fig S4E-F). Because this phenomenon is not a central focus of our study, we did not further pursue the mechanistic reasons why cone opsin fails to rescue rod OS morphology.

7) In Figure 2, Panel G, it looks like the top of the UV cone OSs are cut off, either by the physical or optical section. This is also true for the wt image in Fig 3, panel I and Fig 4B. How is the analysis controlled to ensure that the whole cell is captured? And if these images are correct, then are there changes to OS shape? This is an important point since the length of the OS is a key measurement in the analysis.

Answer:

The reviewer is correct that the UV cone OSs appearing “cut off” do not represent complete outer segments. Such truncation can occur due to sectioning artifacts or physiological processes such as RPE phagocytosis. Importantly, in all quantitative analyses, we measured only intact UV cone outer segments with clearly defined distal tips to ensure accurate assessment of OS length.

We thank the reviewer for this careful observation and apologize for the suboptimal image selection in the original submission. In the revised manuscript, we have replaced these panels with more representative images.

Minor comments:

1) There are no page or line numbers, which makes it difficult to provide clear comments.

2) Intro: The OS is not analogous to a dendrite. Receives signals, but very different structure.

3) Intro: The authors state the "OS resemble a modified cilium". The OS is a modified cilium

4) Intro: "By comparison, the retina of the zebrafish is predominantly cone-based, with approximately 92% cones at the larval stage and about 60% in adults, closely resembling the cone-rich fovea found in humans (Fadool, 2003, Masek, Zang et al., 2023). There is a missing reference here.

Answer:

Thank you for these comments. We have added line and page numbers in the revised manuscript and modified the introduction and added more references in the revised manuscript.

5) More clarification about ages could be used in the text and on the figures. What is the range of ages described as "adult"?

Answer:

In the revised manuscript, we have specified the age range for adult zebrafish in the text and have clearly indicated the specific developmental timepoints in all relevant figures.

6) For Figure 2, need lower magnification panels to show position/organization of the cells within the retina. Panel E needs a scale bar.

Answer:

Thanks. We have included lower magnification panels to show the position and organization of the cells within the retina and have added a scale bar to Panel E (Fig 2).

7) The relationship between width and length of OS doesn't seem to hold for the other species. Are the scales accurate, since the cell size looks massively different between cone subtypes in some o f the fish. Please comment on the length-width ratio in the discussion.

Answer:

Thank you for this comment. We have verified that all scale bars are accurate. While our study primarily focuses on differences in cone outer segment (OS) length, we fully acknowledge that OS width and the length-to-width ratio are equally important morphological parameters.

The length-to-width ratio may serve as a key "morpho-functional index," reflecting how different species balance competing demands—such as sensitivity, spatial resolution, and metabolic efficiency—within their specific ecological niches. The pattern we observed in zebrafish likely represents an efficient solution adapted to its particular visual ecology. In other species, however, natural selection may have shaped markedly different length-to-width ratios to address unique visual challenges—for example, maximizing sensitivity in deep-sea fish or optimizing color and spatial resolution in

species inhabiting brightly lit coral reefs. We have incorporated discussion of this concept into the revised manuscript.

8) Discussion is generally underwhelming, and the comparison to Hebbian plasticity is weak and d oesn't really add anything to the paper. Neural activity of photoreceptors is not activated by light (t he opposite), which should be made clear. And the LTP comparison is nebulous.

Answer:

Thank you for pointing out these issues. We agree with the reviewer that Hebbian plasticity is not an appropriate analogy for photoreceptor outer segment regulation. In the revised manuscript, we have removed this comparison and expanded the discussion to focus on more relevant and mechanistically grounded possibilities that may underlie cone OS morphological regulation.

Reviewer #3: The manuscript by Xu et al. reports the intriguing finding that the morphology (length and width) of the outer segments (OS), the light-sensitive region of photoreceptors, is regulated by the kind of the opsin expressed in the OS and the intensity and wavelength of incident light. The authors used elegant genetics in the zebrafish model to establish this, and also present data that argue for a conservation of this principle in other fish species as well as mammals. Overall, the experiments are well designed, the data are nicely presented, and the manuscript is written in a very accessible style.

Despite the novelty of their findings, the shortcoming of the paper is that the authors do not present any data or much of an argument on how photoreceptor-specific opsins could be regulating OS morphology at the mechanistic level. Since the OS is essentially an exaggerated elaboration of the connecting cilia membrane, could it be that the opsins, which are OS membrane proteins, regulate membrane biogenesis and organization? For instance, the opsins could act through the effectors of ciliary membrane biogenesis like the Rab proteins and Arl13b? While this manuscript is suitable for PLoS Biology as a short article and I am not expecting the authors to now put together some mechanistic data along these lines in the revision, it would nevertheless be worthwhile to include some speculation on the mechanism in the discussion section

Answer:

We sincerely thank you for this insightful comments. We fully agree that understanding the molecular mechanism by which opsins regulate OS morphology is a central and

unresolved question. Although we do not yet have mechanistic data, several plausible possibilities may be considered.

First, different opsins may differ in their trafficking efficiency or transport dynamics through the connecting cilium, which could influence the rate of membrane delivery and OS membrane biogenesis. Second, opsins itself may differentially affect the organization or packing of phototransduction proteins within disc membranes, thereby altering disc architecture or stability. Third, opsins could potentially interact with regulators of ciliary membrane biogenesis, as suggested by this reviewer, to modulate OS elongation or membrane morphology. Finally, we cannot exclude the possibility of feedback signals from the cell body that adjust OS growth based on opsin type or phototransduction state.

In the revised manuscript, we have incorporated a new paragraph in the Discussion section that elaborates on these potential mechanisms and highlights this important direction for future research.

Reviewer #4 (Michel Cayouette and Michael Housset signed their report): In this manuscript, Xu et al. address how cells adopt different morphologies. To explore this very interesting and poorly studied question, they use primarily the zebrafish photoreceptor cells as a model system, taking advantage of the various morphologies displayed by photoreceptor subtypes and ease of genetic manipulations in zebrafish. Specifically, they examine how opsins of different spectral properties and ambient light intensity influences photoreceptor outer segment (OS) morphology. They report a positive correlation between OS length and the peak absorption wavelength of the expressed opsin, with an inverse correlation between OS base width and absorption peak. This relationship appears conserved across vertebrate species, including different fish species and rabbit. The authors also show that sws1 mutant zebrafish exhibit UV cone degeneration, which is rescued by ectopic expression of sws1 (UV), mws3 (green), or lws1 (red) opsins. Strikingly, the OS morphology of rescued cones varies depending on which opsin subtype is expressed, supporting a role for opsin sensitivity in shaping photoreceptor morphology. They also show that thyroid hormone (TH) treatment, which shifts opsin expression in red cones to a longer wavelength red opsin, further elongates the OS, reinforcing the link between spectral tuning and OS morphology. The authors propose a mode in which OS geometry is an adaptive response to differences in photon energy and

opsin activation efficiency, supported by experiments in dim light and comparisons with deep-sea Sebastes schlegelli.

Overall, this is a very interesting paper addressing a key question in neurobiology. The conclusions are largely supported by the results (although see below), the paper is well written, and the figures are clear and generally convincing. This work will be of interest to vision scientists and to the neuroscience community in general. While the study lacks mechanistic insights into how exactly different opsins leads to changes in cell morphology, these results establish a solid base for future investigation in the field and constitute groundbreaking work in this area. There are, however, a few points that should be clarified and some additional experiments that may help better support the conclusions. Specifically, mechanistic clarity is needed regarding the roles of light versus opsin identity, and additional quantitative and optical analyses are needed to strengthen the central claims. These points are detailed below.

Major comments

1) In fish, as well as chicken and xenopus, photoreceptors dynamically elongate or contract in response to light and dark adaptation, but the implication of this phenomenon on the interpretation of the data presented in this paper is not discussed. It is crucial that the authors discuss their findings in the context of retinomotor movements. This background would help distinguish between reversible, transient changes and more stable OS modifications.

Answer:

Thank you for pointing out these issues. We agree that teleost fish, as well as chickens and xenopus, exhibit retinomotor movements in response to light and dark adaptation. This is indeed a phenomenon of great interest to us, and in a separate research project, we are systematically investigating the mechanisms underlying retinomotor movement in rods and cones. We have included partial of these data below (attach a figure here).

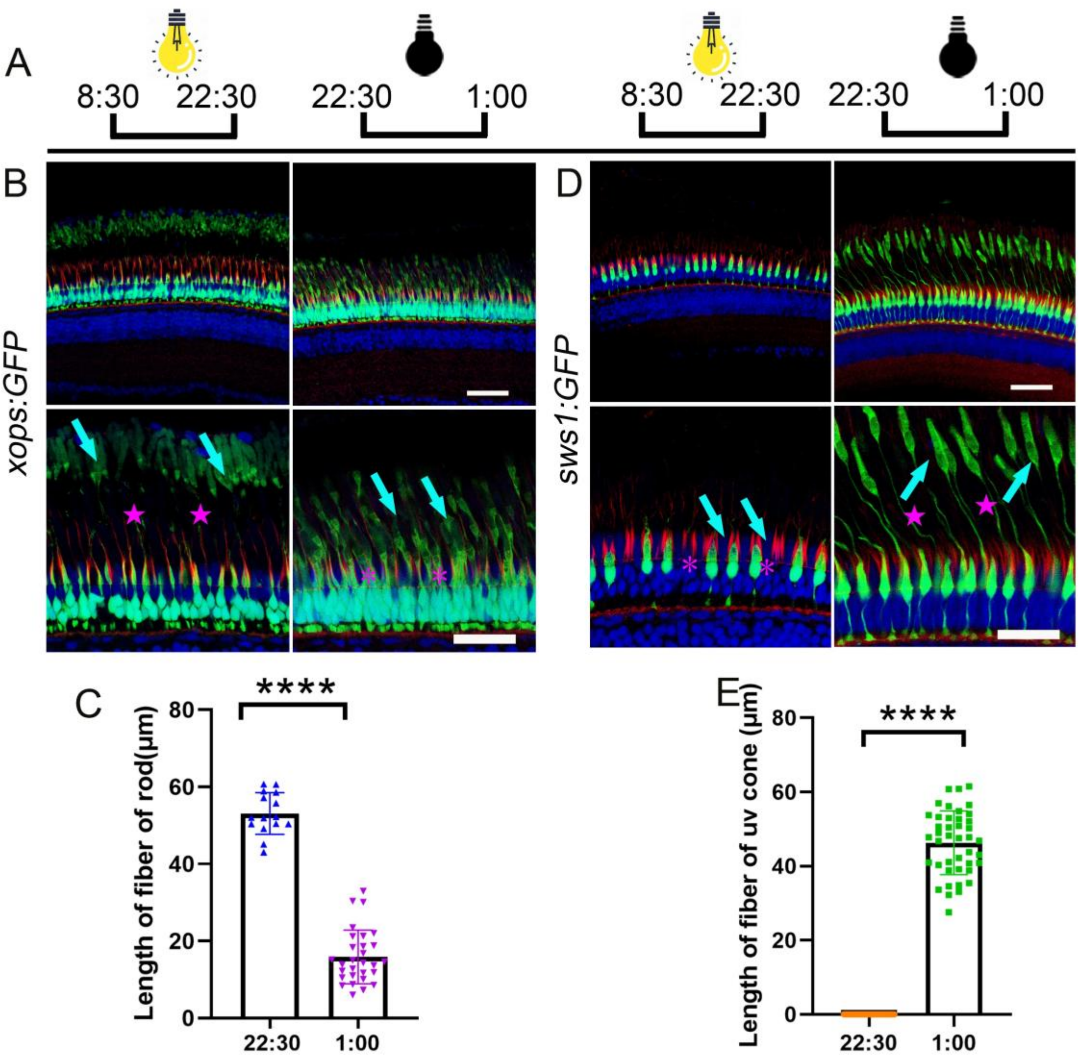

In the present study, we fixed all zebrafish and other teleost retinas during the daytime hours between 12:00 to 17:00. At this daytime, the retinomotor movements were minium and the length of cone outer segments remains consistent, as illustrated in the figure below. This standardized fixation protocol ensures that our measurements reflect stable OS morphology rather than transient light-adaptive changes. We have included these information in the revised manuscript.

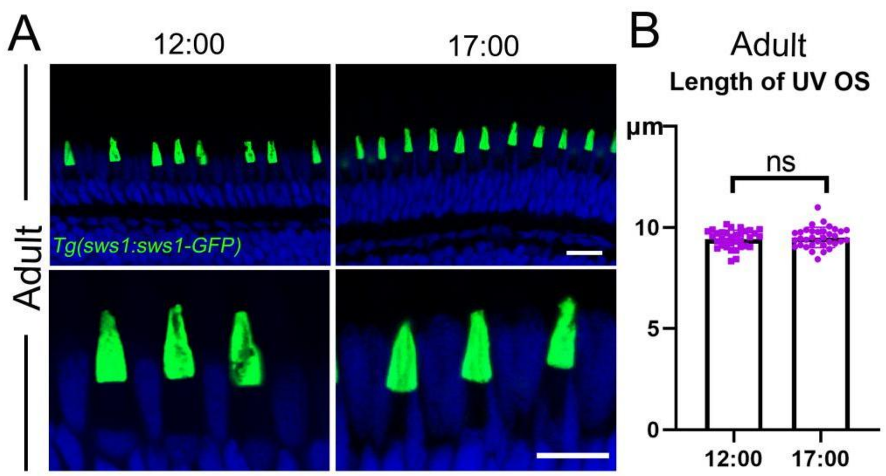

2) While the study convincingly links opsin identity to OS morphology, the interpretation that OS length reflects an adaptation to help increase opsin content is not fully substantiated. OS length and width are not an adequate proxy for opsin quantity because the amount of opsin proteins that can accumulate in OS disk membrane depends on volume. A quantification of OS volume and surface area, ideally supported by 3D reconstructions, would provide a more robust test of the authors' hypothesis of a gain in opsin content in the OS.

Answer:

Thank you for these constructive suggestions. We agree that OS length alone is not a sufficient proxy for total opsin content, as the capacity for opsin accumulation ultimately depends on both membrane surface area and overall OS volume. Following the reviewer's recommendation, we performed 3D reconstructions and quantified outer-segment volume. Our analyses showed that blue-cone OSs have slightly larger volumes than UV-cone OSs. We also quantified OS volume under conditions where OS elongation occurs, including dim-light rearing and ectopic lipid-droplet induction. In both cases, these manipulations increased not only OS length but also OS volume, indicating that the elongation is accompanied by an expansion of overall membrane capacity (see attached figures and also submitted videos).

At the same time, we would like to clarify that a larger OS volume does not necessarily equate to a higher opsin content, unless the comparison is made within the same opsin type. Differences in opsin packing density, disk-membrane spacing, or the structural organization of disks could vary between cone subtypes. Moreover, computational 3D reconstructions may not perfectly capture the true OS volume. For these reasons, we still consider OS length to be a practical and reliable parameter for comparing relative OS changes across different cone types in this study.

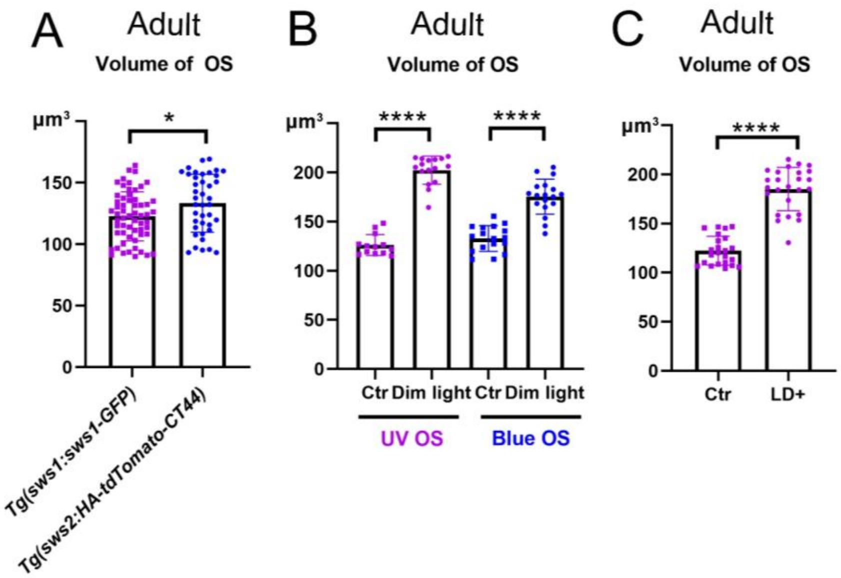

(A) Quantification of UV and blue cone OS volume; (B) Quantification of UV and blue cone OS volume in adult fish following dim-light treatment; (C) Quantification of UV cone OS volume after ectopic lipid droplet expression.

3) The manuscript would benefit from additional data to strengthen the model. Specifically, it remains unclear whether OS shape is intrinsically determined by the opsin protein itself or requires downstream effects of light activation. These aspects could be experimentally disentangled by raising fish in complete darkness and under monochromatic light of different wavelength. Moreover, the intriguing possibility that opsin identity dictates OS width while photon absorption regulates OS length deserves more extensive exploration. The authors could for example put transgenics Tg (sw1 :Mws3) and Tg (sws1 :Sws1) of the sws1 mutant background in total darkness until analysis. If OS shape is directly influenced by the light-mediated activity, as predicted by the authors' model, there should not be any difference between the two conditions, since there is no light to activate the opsins.

Answer:

Thank you for these excellent suggestions. The first reviewer also raised a similar point regarding whether neural activity contributes to regulating outer segment (OS) morphology in the transgenic lines. Following your recommendations, we raised Tg(*sws1:lws1*) fish under different lighting conditions—including monochromatic illumination—and in complete darkness throughout development.

After 1.5 months, we found that UV cones of Tg(*sws1:lws1*) fish reared in complete darkness—conditions under which Lws1 activation was largely inhibited—display OS lengths comparable to wild-type UV cones (Fig. S8). In contrast, UV cones of Tg(*sws1:lws1*) raised under white or red light (550–565 nm) exhibited a robust elongation of their OSs. These results demonstrate that OS elongation requires light-dependent activation of the ectopic opsin, supporting our model that OS length regulation is activity-dependent rather than determined solely by opsin identity.

Interestingly, blue light (410–420 nm) also induced OS elongation, although Lws1 opsin is not expected to be efficiently activated at this wavelength. At this stage, we cannot determine the precise mechanism. One possibility is that the blue LED light source contains minor spectral leakage into longer wavelengths sufficient to activate Lws1. Alternatively, activation of native blue cones may indirectly influence UV cone OS morphology through inter-cone interactions within the retinal network. While these explanations remain speculative, the dark-rearing experiments clearly demonstrate that photoreceptor activity is required for the Lws1-dependent OS elongation in UV cones.

We have included the darkness-experiment data in the revised manuscript. However, the results from the red and blue LED activation experiments were not conclusive, likely due to limitations in the equipment design. Therefore, we prefer not to include these data in the revised text. The corresponding results are shown below.

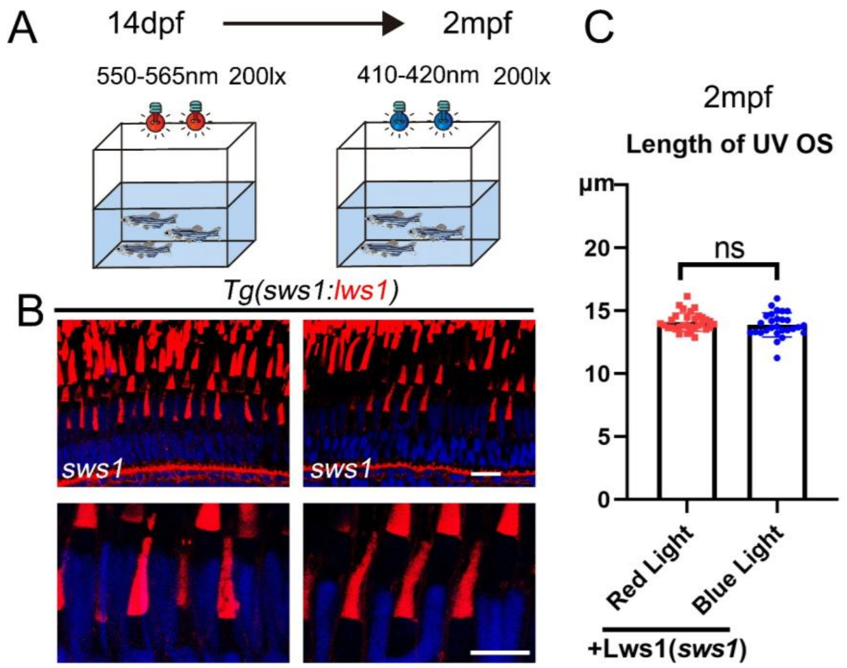

4) The analogy between OS elongation and dendritic arbor refinement is compelling but unclear. The authors should clarify whether dim-light-induced OS elongation occurs during a critical developmental window, as often observed in dendrite refinement, or can be modified post-developmentally by performing experiments in adult animals. If it is not possible to carry out these experiments, this limitation should at least be discussed.

Answer:

Thank you for these constructive suggestions. To address this point, we exposed 5-month-old adult zebrafish to dim-light conditions for one month and observed that cone outer segments in adult animals also exhibited significant elongation (Fig. S8). This result indicates that dim-light–induced OS elongation is not restricted to a critical developmental window but can be modified post-developmentally in mature zebrafish. We have incorporated these new data into the revised manuscript. In addition, as suggested by this and other reviewers, we have softened our discussion regarding the analogy between OS elongation and dendritic arborization.

5) In Figure 1:

* Indicate OS location in panel A.
* Improve contrast for the legend in panel F.
* Specify whether OS width is measured at the base or along the full segment.

6) In Figure 2:

* Panel B: Clarify if the region targeted by the probe lies within, upsteam or downstream of the deleted portion of the sws1 gene.
* Correct the legend: "10 µm in (C-E)" → "10 µm in (C-I)."
* The authors discuss protein levels, but only RNA is quantified in Supplementary Figure 5. Western blotting would be important to confirm protein expression. Also, clarify which genes are being quantified in the figure S5 panel B. Please segregate qPCR done for Sws1 gene and qPCR for Lws1 on different graphics.

Answer:

Thank you for these helpful suggestions. We have modified the figures accordingly. Due to the lack of reliable UV opsin antibodies in zebrafish, we were unable to perform Western blot analysis for UV opsin. To further validate our findings at the protein level, we employed data-independent acquisition (DIA) mass spectrometry, which enables quantitative comparison of UV opsin abundance across samples. The DIA-MS results

showed that *sws1* heterozygous mutants exhibit not only reduced *sws1* mRNA expression but also a decrease in UV opsin protein levels. These new data have been included in the revised manuscript (Fig. S7D), and the original DIA-MS outputs are shown below. Additionally, we have clarified which genes were quantified in Figure S7.

PG_Genes	PG_Protei	PG_Protei	Sequence	wt1	wt2	wt_Mean	Aa1	Aa2	Aa_Mean	P_value
anxa1b	Annexin	Q804H1_DAN	6	27012.2	4999.15	16005.675	9582.45	5570.51	7576.48	8.0037E-05
opn1sw1	Opsin-1, shor	OP1S1_DANF	21	2082400	1600630	1841515	792135	952140	872137.5	0.00015487

7) In Figure 3:

* Change Pseudemys scripta elegans for the correct current taxonomy: Trachemys scripta elegans.

* Panel B: Quantification of OS shape (length and width) is missing for the turtle LWS rescue. Panel C: Consider replacing the current schematic with one showing that TH induce expression of longer wavelength red opsin Lws1 at the expense of the shorter Lws2 at the genomic locus.

Panels F-I: The lipid droplet experiment is technically impressive but lacks clear optical implications. The droplet, likely composed of neutral lipids and localized basally in the myoid, is unlikely to absorb or scatter UV light significantly, contrary to the avian lipid droplet found in birds PRs which contains specific lipids and pigments. Without optical modeling or empirical measurement, the impact on photon flow remains speculative. Conclusions should therefore be toned down and limitations of this approach discussed.

Answer:

Thank you for these constructive suggestions. We have updated the figures accordingly. Regarding the TH-treatment experiments, we would like to clarify that the observed red-shift is not mainly driven by TH-induced differential expression of Lws1 and Lws2. Instead, TH promotes the replacement of the A1 chromophore with the A2 chromophore in LWS opsins, and this A1→A2 chromophore switch is well known to cause a pronounced long-wavelength shift in the absorption spectrum of LWS pigments (Allison, Haimberger et al., 2004, Enright, Toomey et al., 2015). To make this mechanism clearer, we have revised the schematic in Fig 3D to explicitly illustrate how TH induces the chromophore switch and how this conversion directly leads to the red-shifted spectral sensitivity.

For the lipid-droplet experiments, we agree with the reviewer that the optical implications remain speculative. These ectopic lipid droplets are likely composed of neutral lipids, which may differ from the native avian oil droplets. Nevertheless, we think their presence could still influence the path of UV light to some extent, although the effect is likely weaker. Consistent with this notion, the morphological changes in UV-cone OSs were also relatively modest compared with other treatments, such as dim-light rearing or opsin switching. In the revised text, we have clarified these limitations and have softened our interpretation regarding the functional relevance of the lipid droplets.

8) In Figure 4:

* Quantify OS volume and base width in addition to length (or change all quantification to volume measurements, as suggested in point 2 above).

* Clarify how the OS tip was defined in overlapping WGA-labeled double cones.

* Address potential morphological artifacts due to delayed fixation in wild-caught specimens; consider using a control feature (e.g., nuclear diameter or IS length) for normalization.

Answer:

Thank you for these valuable suggestions. In response, we performed 3D reconstructions of UV-cone and blue-cone outer segments from adult zebrafish following dim-light treatment (see previously response).

Regarding the definition of the OS tip in double cones: we typically identify the tip as the distal-most point of the WGA signal. In instances where the two double-cone OSs exhibit substantial lateral overlap such that their distal ends cannot be reliably separated in a single 2D plane, we took advantage of the full 3D Z-stack to trace each OS shaft to its individual terminus. We have clarified this in the Methods section.

Concerning potential morphological artifacts caused by delayed fixation in wild-caught specimens: we would like to emphasize that all teleost samples—both farm-reared and wild-caught—were processed using comparable fixation and immunostaining procedures, and the wild samples were not subjected to prolonged pre-fixation intervals. To further address this concern, we additionally quantified inner segment (IS) length of double cones as an internal control. IS length was comparable between cultured and

wild specimens, suggesting that fixation-related artifacts are unlikely to account for the observed OS differences. We have mentioned the fixation methods in the revised version.

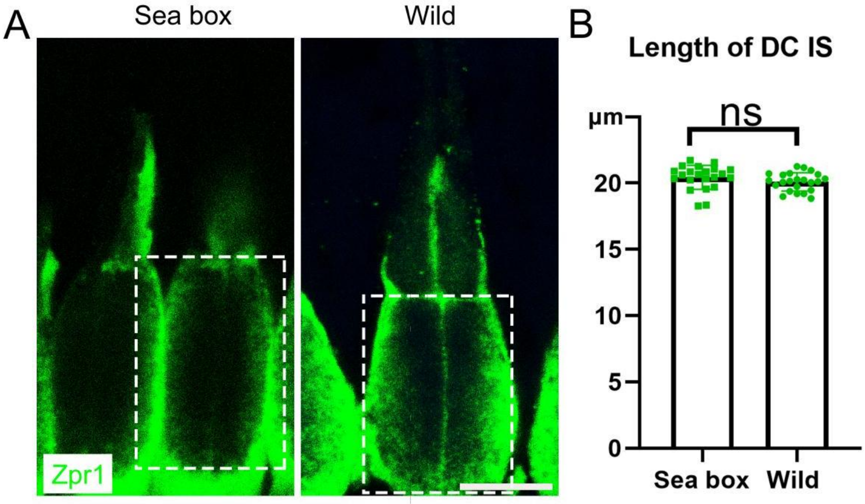

9) In Figure 5: The model presented in not clear. The authors are encouraged to provide a refined, more self-explanatory figure. The position of cones within the retinal layers could be integrated to the model as well as potential effects of wavelength-dependent penetration in the tissue. If additional data can be obtained to distinguish between an intrinsic role of light and opsin protein species in OS width and length, as suggested in point 3 above, this should be considered in the model.

Answer:

Thank you for this helpful suggestion. We have substantially revised Figure 5 to improve clarity and self-explanatory value.

Minor comments

* Regarding the failure to rescue sws1 mutants with tagged opsins, please include a schematic of tag placement and fusion constructs. The C-terminal domain of rhodopsin contains essential trafficking signals, particularly the final 15 amino acids. If this is disrupted by the tag, mislocalization of opsin could explain the lack of rescue. This limitation should be discussed or excluded experimentally.

Answer:

Thanks for these comments. We would like to emphasize that the construct used in Tg(*sws1:HA-tdTomato-CT44*) contains only the C-terminal 44 amino acids of rhodopsin rather than the full-length protein. This C-terminal fragment is sufficient to target fluorescent proteins to OS membrane (Tam, Moritz et al., 2000), but it lacks the

seven-transmembrane core required for proper phototransduction and OS morphogenesis. Therefore, it is not expected to rescue the structural defects observed in *sws1* mutants.

Unexpectedly, the Tg(*sws1:sws1-GFP)* transgene, in which full-length Sws1 is fused to GFP, also failed to rescue the mutant phenotype, despite the fusion protein being correctly targeted to the OS (Fig. S2H-I). As the reviewer suggests, this is likely due to disruptions caused by the C-terminal GFP tag. The C-terminus of opsins contains essential motifs for downstream signaling and G-protein interaction; C-terminal tagging may interfere with these processes, thereby impairing Sws1 function even when localization is preserved. We have now included this information in the revised text.

In Figure S6H, expression of long-wavelength opsins appears to reduce inner segment (IS) length. Please mention impact of opsin on IS length and discuss whether this reflects coordinated regulation of IS and OS dimensions. Also, the limited rescue by Lws1 compared to rhodopsin raises concerns about expression efficiency or structural compatibility. please clarify what happens to non-rescued rods.

Answer:

Thank you for these insightful comments. We would like to clarify that the length measured in Figure S6H represents the distance between the base of the rod OS and the outer plexiform layer (OPL), rather than the inner segment (IS) length. This parameter was intentionally chosen to illustrate the altered vertical positioning of rod OSs upon ectopic Lws1 expression. In wild-type zebrafish, rod somata typically extend longer apical processes, allowing their OSs to occupy a more distal position during daytime imaging. When Lws1 is ectopically expressed in rods, this distance is markedly reduced, indicating that these rods adopt a more basal OS position.

Regarding the limited rescue efficiency: although Lws1 expression prevents the complete loss of rod OSs, the rescued OSs appear sparse compared with the densely packed OS layer in wild-type retinas. Consistently, Lws1 fails to fully prevent rod degeneration even at larval stages, whereas rhodopsin provides robust rescue. This suggests that Lws1 is not structurally or functionally equivalent to rhodopsin for

maintaining rod OS integrity, despite its correct localization. We have included and discussed these issues in the revised version (Fig S4E-F).

* Clearly state "n" values (number of cones vs. number of animals) for each experiment.

* Indicate developmental stage and retinal region for all quantifications.

* In several figures, the authors used a student's t-test for comparison between multiple conditions, which is not appropriate statistics. ANOVA with post hoc corrections must instead be used.

Answer:

Thank you for pointing out these important issues. In the revised manuscript, we have carefully reviewed all quantification panels and have now clearly stated the n numbers for each experiment. We added information about the developmental stage and the exact retinal region used for each quantification. We have also re-evaluated the statistical methods across all figures and have replaced inappropriate Student's t-tests with ANOVA or Kruskal-Wallis test for comparisons involving more than two groups. The revised statistical approaches are now indicated in the figure legends and methods section.

References

Allison WT, Haimberger TJ, Hawryshyn CW, Temple SE (2004) Visual pigment composition in zebrafish: Evidence for a rhodopsin-porphyropsin interchange system. *Visual neuroscience* 21: 945-52

Enright JM, Toomey MB, Sato SY, Temple SE, Allen JR, Fujiwara R, Kramlinger VM, Nagy LD, Johnson KM, Xiao Y, How MJ, Johnson SL, Roberts NW, Kefalov VJ, Guengerich FP, Corbo JC (2015) Cyp27c1 Red-Shifts the Spectral Sensitivity of Photoreceptors by Converting Vitamin A1 into A2. *Current biology : CB* 25: 3048-57

Fariss RN, Anderson DH, Fisher SK (1990) Comparison of photoreceptor-specific matrix domains in the cat and monkey retinas. *Experimental eye research* 51: 473-85

Garlipp MA, Nowak KR, Gonzalez-Fernandez F (2012) Cone outer segment extracellular matrix as binding domain for interphotoreceptor retinoid-binding protein. *The Journal of comparative neurology* 520: 756-69

Tam BM, Moritz OL, Hurd LB, Papermaster DS (2000) Identification of an outer segment targeting signal in the COOH terminus of rhodopsin using transgenic Xenopus

laevis. *The Journal of cell biology* 151: 1369-80
