# Supplementary material for: Light intensity and opsin sensitivity shape the morphology of cone photoreceptor outer segments
Source: PLoS Biol. 2026 Feb 18;24(2):e3003654. doi: 10.1371/journal.pbio.3003654 (PMC12915902; doi:10.1371/journal.pbio.3003654)
Supplement: Attachment — Submitted filename: PBIOLOGY-D-25-02009 Response to Reviewers 2.docx [file pbio.3003654.s014.docx]

Reviewer remarks:

Reviewer #1 [Juan Angueyra]: The authors addressed our comments satisfactorily, including reassessing parallels with Hebbian plasticity, improving description of methods and structural rearrangements to improve readability. The concerns of other reviewers have also been sufficiently addressed. We believe these edits have improved the paper and strengthened their models to explain the mechanisms that may dictate the structure of photoreceptor outer segments in relation to the environment. We only have two minor suggestions for revisions of the specific interpretations that won't require any additional experiments, and that we leave to the authors discretion.
First, the following paragraph is still inaccurate and/or confusing:
"Light energy acquisition depends on two main factors: the energy of individual photons and the number of photons captured by photopigments. Reducing photon energy (e.g., using longer wavelengths) or limiting photon capture (e.g., through additional lipid droplets or under dim-light conditions) lowers the probability of opsin activation".
As we previously stated, just using longer wavelengths does not guarantee less opsin activation, which this sentence seems to imply. If the authors intended "to emphasize that, for a given opsin at its optimal wavelength, higher-energy photons generally have a higher probability of causing isomerization than lower-energy photons at their respective optimal wavelengths", this should be incorporated in the manuscript, although we still find even this explanation confusing. Since the "number of photons captured by photopigments" depends on photon energy/wavelength AND spectral sensitivity of the photopigment, this phrasing hides an important logical step. I would argue that it's clearer to say that photon capture depends on two main factors: the total number of photons and the relation between the spectral composition of these photons and the spectral sensitivity of the photopigment. Lowering opsin activation requires decreasing the number of photons that reach the opsins or decreasing the spectral overlap between the stimulus and the opsin.
Second, it might be worth elaborating or clarifying a bit more on what the authors consider as the most likely biological implementation of how "neural activity" leads to changes in OS length. As briefly mentioned, transducin activation and glutamate release in photoreceptors are inversely related, so that "when fish are raised in darkness", there is indeed minimal transducin activation but glutamate release by photoreceptors is at its highest, which could be considered maximal neural activity? Do the authors assume that this could be processed through circuits in the ON pathway, which flips the polarity of signals? Is that more likely than a cell-autonomous mechanism depending directly on the number of activated transducins in individual cones? Disclosing glutamate release in the final model figure may make this explicit for readers.
Answer:
Thank you for these suggestions.

First, we would like to clarify that our intention was to state that shorter-wavelength photons, which carry higher energy, may have a higher probability of activating their corresponding opsins (e.g., UV light activating Sws1 opsins) compared with longer-wavelength photons with lower energy activating their respective opsins (e.g., red light activating Lws1 opsins). We did not intend to suggest that switching between different wavelengths of light alters the activation efficiency of a given opsin protein. We apologize for this lack of clarity in the original text and have revised the Discussion accordingly in the revised manuscript.

Second, we agree with the reviewer that signals from bipolar cells may be involved in the feedback regulation of cone OS length. However, this possibility remains speculative at present, and it is unclear whether ON or OFF bipolar cells participate in this process. In addition, feedback signals intrinsic to photoreceptors themselves may also contribute to OS length regulation. We have briefly mentioned these potential mechanisms in the revised version.

Reviewer #2: The revised paper is strong and acceptable for publication, with a couple of suggested minor changes.

1. The scale bar in Fig4 labelled as "10 cm in (D)" should be "10 cm in (I)".
Answer:
Thank you. We have corrected this in the revised manuscript.

2. The WGA staining in the manuscript is clearly labelling cones. However, this is unusual. I'm unclear if that's a result of a different WGA preparation, more concentrated solution, or imaging parameters. It should be mentioned in the manuscript that WGA is typically used a rod OS marker, but that the researchers found it could be adapted for cone labelling. Otherwise, it will confuse the reader. Also, it appears in many of the panels that WGA fails to label the top portion of the UV cone OS. If so, this should be mentioned.
Answer:
Thank you for these suggestions. We have added this information to the revised manuscript. The absence of WGA staining in the apical portion of the UV cone OS may be due to sectioning artifacts, as noted in our previous response.

4. The panels and labels in Fig S6 are very small. The figure could be reorganized to enlarge panels.
Answer:
Thanks, we have modified this Figure.

Reviewer #4 [Michel Cayouette and Michael Housset]: The authors have done a wonderful job addressing our comments both with text changes and new experiments. We have no further comments. Congratulations on a very interesting study.

We sincerely appreciate the reviewer's recognition and valuable suggestions.
